# Light-Responsive Hydrogels for Soft Robotics: From Multiscale Actuation Mechanisms to Programmable Locomotion and Functional Applications

**DOI:** 10.3390/gels12100940

**Published:** 2026-10-20

**Authors:** Kun Li, Weize Yuan, Zhihao Yang, Yong Tian, Wenguang Yang, Ruiqian Wang

**Affiliations:** 1 School of Electromechanical and Automotive Engineering, Yantai University, Yantai 264005, China; 17866857776@163.com (K.L.); 15258004557@163.com (W.Y.); 18764733206@163.com (Z.Y.); ytu_yangwg@163.com (W.Y.); 2 School of Machinery and Power Engineering, Weifang University, Weifang 261000, China; tianyongjizi0806@163.com; 3 Shandong Key Laboratory of Intelligent Manufacturing Technology for Advanced Power Equipment, Weifang 261000, China

**Keywords:** light-responsive hydrogels, smart polymer networks, soft robotics, photothermal actuation, photochemical response, multimodal locomotion, biomedical hydrogel microrobots

## Abstract

Light-responsive hydrogels combine large reversible deformation with remote, non-contact, and spatially selective control, providing a versatile material basis for untethered soft robots. This review critically examines recent progress from photothermal conversion, molecular photoswitching, network photochemistry, and interfacial regulation to fabrication, structural design, locomotion, sensing, and biomedical operation. Comparative analysis indicates that robotic performance is governed not by hydrogel responsivity alone, but by the coupling of network strain, heat and solvent transport, programmed anisotropy, body geometry, and environmental interactions. Photothermal systems provide efficient remote activation and broad material compatibility, yet their speed and operating stability remain constrained by thermal dissipation and water migration. Photochemical systems offer finer spatial and wavelength selectivity, but optical penetration, chromophore fatigue, chemical reversibility, and auxiliary reagents remain important limitations. Bilayers and continuous gradients reliably transform differential swelling into bending, whereas anisotropic networks, additive manufacturing, and femtosecond-laser processing enable rapid actuation, addressable joints, and programmable three-dimensional architectures. Across crawling, rolling, jumping, swimming, and multimodal operation, net motion requires spatiotemporal symmetry breaking through friction, self-shadowing, topology, or fluid coupling. The review therefore distinguishes directly deformation-driven robots from hybrid platforms in which light regulates adhesion, charge, reconfiguration, or fluid flow while magnetic or electric fields provide propulsion. Emerging applications include intrinsic strain sensing, cargo manipulation, localized cellular stimulation, and three-dimensional drug screening. Future progress requires standardized performance metrics, improved optical delivery, faster mass transport, reduced thermal exposure, long-term cyclic stability, and biodegradable material systems.

## 1. Introduction

A central objective of soft robotics is to construct machines from compliant materials that can adapt to complex environments, establish safe contact with surrounding objects, and perform diverse modes of locomotion and manipulation through continuous body deformation. Unlike conventional robots assembled from rigid links, mechanical joints, and concentrated actuators, soft robots often distribute deformation and mechanical function throughout their material bodies. In this context, hydrogels have emerged as important candidate materials for soft actuators and robotic systems because their three-dimensional hydrophilic polymer networks combine high water content, low elastic modulus, large reversible deformation, permeability, and sensitivity to environmental changes [1,2]. By absorbing or expelling water, a hydrogel can alter its volume, shape, and mechanical properties, allowing sensing, energy conversion, and mechanical response to occur within the same material system. Temperature, pH, ionic strength, electric fields, magnetic fields, and chemical species can all regulate hydrogel behaviour, but these stimuli differ in their capacity for remote addressing, local control, and system integration. Light can deliver both energy and control information without establishing a mechanical connection to the robot and can be programmed through wavelength, intensity, irradiation position, beam geometry, and temporal sequence. Light-responsive hydrogels have therefore become an important material platform for untethered soft robots and micromachines [3,4,5,6,7]. During the past decade, the field has progressed from simple bending or contraction under uniform illumination to self-oscillating phototactic swimmers, light–magnetic hybrid locomotion, and complex deformation generated by patterned multimaterial structures [8,9,10]. Recent studies have further demonstrated independently steerable microcrawlers, adaptive soft robotic optical systems, material-embedded logic operations, and integrated actuation–sensing functions [11,12,13,14]. These advances indicate that the principal research question is shifting from whether a hydrogel can undergo photo-induced deformation to how optical energy can be converted across different length scales into repeatable, directional, and task-relevant robotic behaviour.

However, “light-driven” does not denote a single energy-conversion process. Even when different hydrogel robots exhibit similar bending, contraction, or displacement, the physicochemical processes governing their deformation rate, force output, and operational stability may differ fundamentally. Photothermal conversion is currently one of the most widely used actuation routes and generally involves optical absorption, non-radiative energy dissipation, local heating, modification of polymer–solvent interactions, and mechanically constrained macroscopic deformation. In a representative thermoresponsive network, heat generated by an optical absorber may induce polymer-chain conformational changes, water migration, melting of crystalline domains, or a local pressure increase. The performance of a photothermal actuator therefore cannot be evaluated solely from its optical absorption or maximum temperature; it also depends on optical penetration depth, heat conduction, phase-transition temperature, solvent-transport distance, network thickness, and heat exchange with the environment. Lee et al. incorporated magnetite nanoparticles into comb-type PNIPAAm hydrogels, in which mobile graft chains shortened the effective diffusion distance and accelerated light-induced contraction and recovery [15]. Ni et al. combined photothermal conversion with a dynamically crosslinked and mechanically programmable PNIPAM network. Rapid thermally induced conformational changes of the oriented polymer chains enabled oscillation frequencies of up to 1.7 Hz and allowed the same robot to be reprogrammed for swimming, crawling, and rotation [16]. Wang et al. established a different photothermal pathway by incorporating reduced graphene oxide into a poly(stearyl acrylate-co-methacrylic acid) hydrogel. Local photothermal heating melted crystalline domains formed by the stearyl-acrylate side chains and reduced the apparent density of the hydrogel below that of water. Cooling restored crystallization and increased the density, enabling light-controlled floating, sinking, swimming, rolling, somersaulting, and walking [17]. Li et al. further used photothermally generated water-vapour bubbles together with elastic energy stored in a poly(sodium acrylate) network to produce rapid jumping, rolling, and obstacle crossing in air [18]. These studies demonstrate that photothermal actuation is not a single mechanism with a universal rate-limiting step. Depending on material composition and architecture, the response may be controlled by heat diffusion, polymer-chain rearrangement, water migration, crystalline phase conversion, or local pressure release. Distinguishing these processes is essential for establishing appropriate performance metrics and comparing different robotic systems.

**Figure 1:**
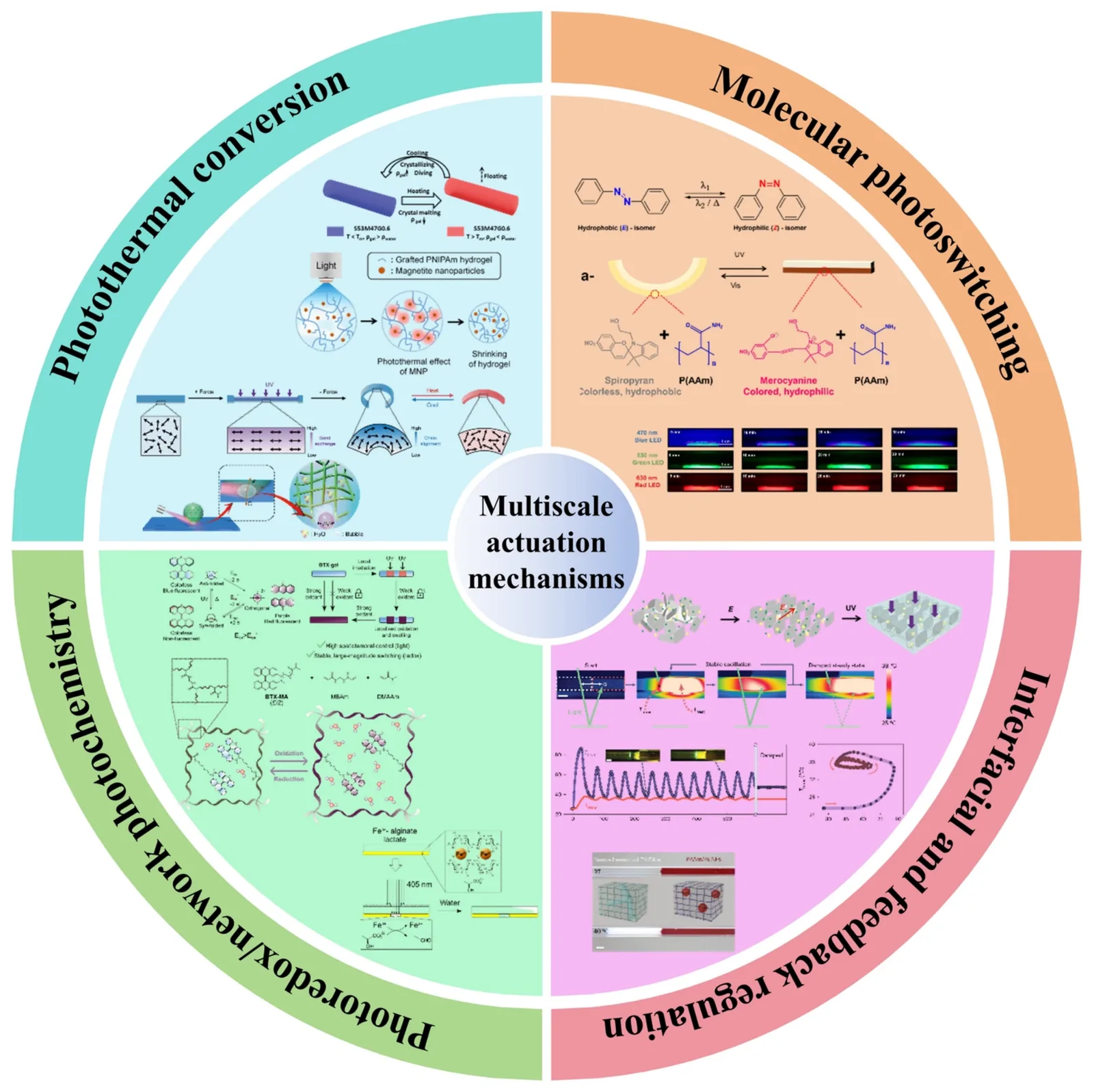
Multiscale actuation mechanisms of light-responsive hydrogel soft robots. The mechanisms are organized clockwise from the upper-left quadrant. Photothermal systems convert absorbed light into localized heat, subsequently activating a volume-phase transition, crystalline transition, density variation, or bubble-mediated impulse [15,16,17,18]. Molecular photoswitching changes the configuration, polarity, or charge state of chromophores and thereby modifies polymer–solvent interactions and network swelling [22,23]. Photoredox and network-photochemical systems alter the redox or charge state of covalently incorporated photoswitches or cleave ionic crosslinks, thereby regulating network hydration, swelling, degradation, and spatial patterning [26,27]. Photoredox and network-photochemical processes regulate crosslink density, coordination state, degradation, or spatial patterning through light-induced chemical reactions [24,25].

Molecular photoswitching provides a more direct route for translating photon absorption into changes in network thermodynamics. Photochromic groups such as spiropyran and azobenzene reversibly change their molecular configuration, polarity, charge distribution, or hydrophilicity under selected wavelengths. When covalently incorporated into a hydrogel, these molecular events modify osmotic pressure and polymer–solvent affinity, leading to macroscopic swelling or contraction without requiring substantial bulk heating. The molecular structure of spiropyran derivatives and the proton-donating environment of the network can be adjusted to improve the operating rate of light-controlled hydrogel valves [19]. Oppositely responding spiropyran layers have also been combined so that photoexpansion of one layer and photocontraction of the other reinforce the bending moment, supporting predictable origami-like transformations and locomotion [20]. Continuum modelling of related photoactive folds has shown that light intensity, optical attenuation, active-domain width, and sheet geometry jointly determine the attainable folding morphology [21]. In Figure 1, azobenzene-functionalized PNIPAAm illustrates how reversible *E*–*Z* isomerization can shift and split the lower critical solution temperature of a hydrogel [22], whereas spiropyran–polyacrylamide bilayers demonstrate how photoisomerization-induced solvent affinity can be converted into reversible bending [23]. Weber et al. introduced a polymerizable bisthioxanthylidene switch into a hydrogel network and used light to gate its two-electron oxidation. Formation of the dicationic state increased the charge density, hydration, and swelling of the network, allowing spatially selective deformation. Therefore, this system should be described as redox-controlled modulation of polymer hydration and swelling rather than as direct photochemical regulation of crosslink density [24]. Bruchet et al. showed that photochemical reduction of Fe(III) crosslinks in alginate enabled localized degradation and spatial patterning [25]. Molecular switching generally provides wavelength selectivity and chemical programmability, but its robotic implementation must still address optical penetration, switching quantum yield, chromophore fatigue, incomplete back-isomerization, and the mechanical dilution caused by low concentrations of photoactive groups.

Macroscopic robotic behavior finally depends on how deformation is coupled to the surrounding environment. A reversible volume change alone produces no net displacement unless temporal or spatial symmetry is broken through anisotropic structure, asymmetric illumination, contact mechanics, hydrodynamic interaction, or feedback. Zhu et al. demonstrated this principle using an anisotropic muscle-like hydrogel in which a scanning light beam simultaneously generated localized isochoric deformation and altered friction against the substrate; coordination of these two responses produced crawling, walking, and turning without separately controlled actuators [26]. Zhang et al. advanced the same concept from open-loop stimulation to feedback-regulated dynamics by thermally coupling light-absorbing and thermoresponsive hydrogels, producing homeostatic oscillations, dynamic color changes, cargo transport, and dissipative signal transduction under constant illumination [27]. More recently, photothermofluidic coupling has enabled an underwater hydrogel vehicle to establish a self-regulated asymmetry and steer toward incident light [28]. Hybrid hydrogel swimmers have likewise converted light-induced local stiffness changes into autonomous phototaxis during magnetically driven flapping [29], while artificial phototropic materials have used an internal photothermal–mechanical feedback loop to align continuously with the direction of illumination [30]. Figure 1 thus emphasizes a hierarchy extending from photon absorption and molecular conversion to network strain, interfacial force generation, and system-level regulation. This multiscale perspective provides the basis for the following sections, which examine the actuation mechanisms, fabrication methods, structural strategies, locomotion modes, multimodal behaviors, and application-oriented functions of light-responsive hydrogel soft robots.

Against this background, the present review examines light-responsive hydrogel soft robots through a multiscale sequence extending from optical input and molecular or network response to structural strain, interfacial interaction, locomotion, and task execution. The first objective is to distinguish photothermal conversion, molecular photoswitching, network photochemistry, and interfacial or feedback regulation according to the primary event that follows photon absorption, rather than grouping systems solely according to their final deformation. The second objective is to compare how bilayers, continuous gradients, anisotropic networks, programmable monolayers, topological bodies, and lattice architectures transform local volume changes into bending, twisting, folding, and non-reciprocal motion and to examine the roles of three-dimensional printing, digital-light processing, and femtosecond-laser fabrication in producing these structures. The review then connects material response with crawling, peristalsis, jumping, rolling, rotation, swimming, and multimodal operation, with particular attention to friction regulation, scanning illumination, self-shadowing, topological constraints, and fluid coupling. Finally, sensing, cargo manipulation, cellular stimulation, and drug-screening applications are discussed together with persistent challenges involving optical attenuation, heat and mass transport, thermal safety, cyclic durability, performance standardization, and biodegradability. Figure 1 summarizes these actuation pathways across multiple length scales. By connecting material chemistry, structural design, and robotic function, this review aims to provide a consistent basis for comparing different light-responsive hydrogel systems and a clearer framework for task-oriented soft-robot design and functional integration.

This article was prepared as a structured narrative review rather than as a systematic meta-analysis. The literature was screened using combinations of descriptors related to photoresponsive, light-responsive, photothermal, photochemical, photoswitchable, and plasmonic hydrogels together with actuator, soft robot, microrobot, locomotion, and manipulation. Primary studies were prioritized when the hydrated polymer network served either as the strain-generating component or as an essential light-controlled element of a hybrid robotic system and when the study reported identifiable information on optical input, deformation, response or recovery time, locomotion, force generation, or task performance. Review articles were used principally to establish background and terminology. Studies based exclusively on dry photoactive polymers, systems in which the hydrogel had no identifiable mechanical or control function, and repeated reports of the same platform without a distinct mechanism, architecture, or robotic task were not treated as core examples. No rigid publication-year cutoff was imposed: foundational studies were retained when they established a distinct actuation principle, whereas recent primary studies were emphasized when evaluating current fabrication, control, and application capabilities. To limit concentration on individual research groups or material classes, the final literature set was examined across photothermal conversion, molecular photoswitching, network photochemistry, and interfacial or feedback regulation, as well as across carbon-based, MXene, magnetic, semiconductor, rare-earth, plasmonic-metal, and molecular-chromophore systems. Hybrid platforms were included only when the respective contributions of the light-responsive hydrogel and the accompanying magnetic, electrical, or fluidic field could be distinguished.

## 2. Actuation Mechanisms of Light-Responsive Hydrogel Soft Robots

Building on the multiscale framework introduced above, this section compares actuation mechanisms according to the first physicochemical event following photon absorption and the transport process that governs the resulting deformation. Photothermal systems are controlled primarily by light-to-heat conversion coupled to thermal and solvent transport, whereas photochemical systems alter molecular configuration, charge, or network connectivity more directly. This distinction provides the basis for comparing their response rate, spatial addressability, reversibility, and operating constraints.

### 2.1. Photothermal Conversion and Phase Transitions in Smart Polymer Networks

Rather than treating photothermal conversion as a single material property, this section focuses on the coupled transport processes that determine whether absorbed optical energy becomes useful mechanical deformation. The temperature field reflects volumetric heat generation, conduction within the gel, and heat exchange with the surroundings, whereas the resulting strain additionally depends on optical attenuation, actuator thickness, water content, and illumination geometry. Consequently, similar photothermal absorbers may produce markedly different response times and deformation amplitudes in different hydrogel architectures, because water simultaneously serves as a heat-transfer medium, a mobile participant in the phase transition, and a source of poroelastic resistance [31,32,33].

Thermoresponsive poly(*N*-isopropylacrylamide) (PNIPAM) is frequently used as the active matrix. Below its lower critical solution temperature (LCST), hydrogen bonding between the amide groups and water stabilizes an expanded network. Heating above the LCST weakens polymer–water interactions and promotes hydrophobic association of the isopropyl groups, causing a coil-to-globule transition and expulsion of water. Because neat PNIPAM does not efficiently absorb near-infrared light, photothermal fillers are commonly introduced to create a local heat source. Representative fillers include reduced graphene oxide (rGO), carbon nanotubes, MXenes, iron oxide nanoparticles, metallic nanostructures, and semiconductor or rare-earth oxide particles [34,35,36].

Plasmonic metal nanostructures constitute a distinct alternative to broadband carbon-based photothermal absorbers. In gold nanorods, the longitudinal localized surface plasmon resonance can be adjusted through particle geometry, allowing the principal absorption band and the heat-generation region to be matched to a selected near-infrared wavelength. Kim et al. incorporated gold nanorods with an aspect ratio of 4.12 and an absorption maximum near 880 nm into a PNIPAAm/PAAm bilayer; the actuator completed ring-like bending within 1 min under an irradiation intensity below 1.0 W cm^−2^ [37]. At the microscale, Özkale et al. assembled thermoresponsive pNIPMAM-coated gold nanorods into optomechanical actuators and integrated them with PEGDA hydrogel mechanisms. The resulting elements produced a relative stroke of up to 0.3 and stress of up to 10 kPa, while laser addressing provided submicrometre spatial resolution and millisecond temporal resolution [38]. Gold nanorods stabilized with thiol-terminated PEG have also been incorporated into printable PNIPAM-based inks, enabling spatially localized near-infrared deformation in four-dimensional printed structures, although the reported shape-morphing experiments were conducted under non-immersed conditions [39]. Compared with carbon fillers, plasmonic transducers offer stronger spectral tunability and highly localized heating at relatively low particle contents. Their performance, however, is more sensitive to particle aspect-ratio distribution, orientation, aggregation, interparticle coupling, and changes in the surrounding refractive index during hydrogel swelling. Surface-ligand stability, noble-metal cost, local thermal hotspots, and long-term retention must also be considered. Plasmonic and carbon-based absorbers should therefore be regarded as complementary rather than hierarchically superior material classes: the former are particularly attractive for spectrally addressable microscale actuation, whereas the latter generally provide broadband absorption and comparatively straightforward implementation in larger actuators.

**Figure 2:**
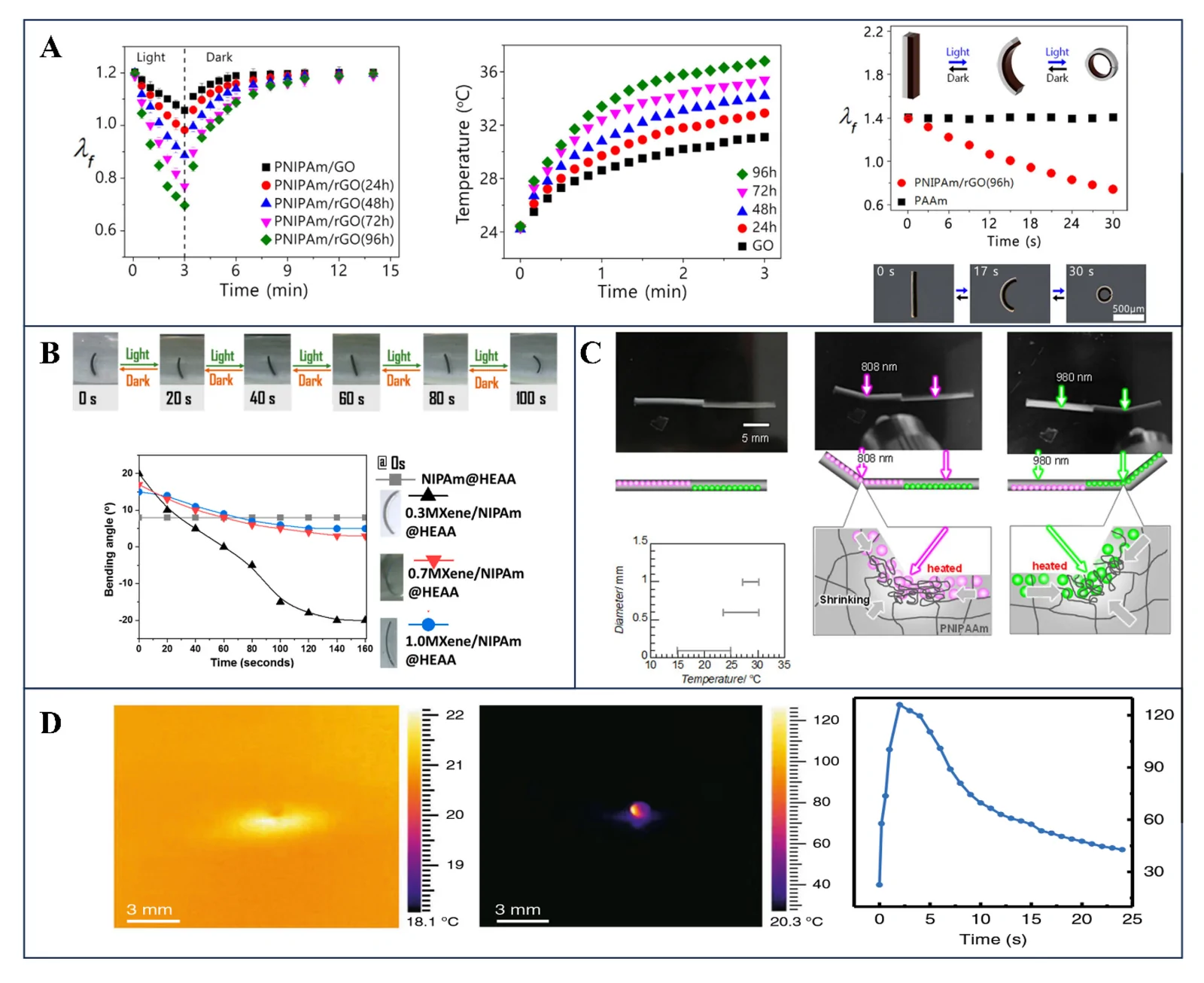
Photothermal conversion–phase transition–deformation mechanisms in light-responsive hydrogel actuators. (**A**) Photothermal heating, reversible dimensional variation, and bending of an rGO-containing PNIPAM/PAAm bilayer actuator [40]. (**B**) Light-induced bending of a Ti_3_C_2_T_x_ MXene/PNIPAM–HEAA bilayer as a function of MXene loading and irradiation time [41]. (**C**) Wavelength-selective actuation of thermoresponsive hydrogels containing Nd_2_O_3_ and Yb_2_O_3_ photothermal particles under 808 and 980 nm irradiation [42]. (**D**) Local photothermal heating of an iron oxide nanoparticle-containing hydrogel and the temperature rise responsible for water vaporization and bubble-mediated impulsive deformation [18]. The panels were adapted and assembled from the cited open-access articles.

Figure 2A illustrates an rGO-containing PNIPAM hydrogel laminated with a passive polyacrylamide layer. Chemical reduction of graphene oxide within the hydrogel improves optical absorption while limiting aggregation. Under illumination, the active PNIPAM/rGO layer heats, contracts, and develops a strain mismatch relative to the passive layer, resulting in reversible bending. The dependence of temperature, length ratio, and bending response on the reduction conditions also demonstrates that the concentration and photothermal state of the filler must be optimized rather than maximized indiscriminately [40]. Excessive absorber loading can reduce light penetration and concentrate heat near the illuminated surface, producing steep but shallow thermal gradients.

The same bilayer principle is implemented with Ti_3_C_2_T_x_ MXene in Figure 2B. In this actuator, an MXene/PNIPAM layer is coupled to a comparatively non-responsive hydrogel layer. MXene nanosheets provide broadband absorption and efficient thermal transport, while their concentration controls both the heating rate and the mechanical properties of the active layer. The observed dependence of bending angle on MXene content shows that photothermal conversion, network stiffness, and interfacial strain transfer cannot be optimized independently [41]. More elaborate control can be achieved by orienting or spatially distributing MXene nanosheets. Electrically induced anisotropy and mask-assisted polymerization, for example, have been used to define predetermined bending directions and sequential gripper motions under localized near-infrared irradiation [35].

Spectral selectivity provides another level of control. Figure 2C shows a thermoresponsive hydrogel containing Nd_2_O_3_ and Yb_2_O_3_ particles, which exhibit preferential absorption near 808 and 980 nm, respectively. Selective irradiation locally heats the corresponding particle-containing region above the phase-transition temperature, allowing different sections of a single actuator to be addressed by wavelength rather than by moving the light spot [42]. This strategy reduces the need for complex external beam positioning, although its operating window depends on the initial temperature of the aqueous environment. If the gel temperature is far below the LCST, high optical power is required; if it is too close to the LCST, thermal diffusion may activate adjacent regions and degrade wavelength selectivity.

The translation from phase transition to macroscopic deformation requires mechanical asymmetry. In a bilayer, differential strain between the active and passive layers generates curvature. In a compositionally graded or anisotropic network, the same function is provided by a continuous variation in crosslink density, filler concentration, fibre orientation, or porosity. Continuous gradients can distribute interfacial stress more effectively than sharply separated layers and reduce the likelihood of delamination. Recent gradient-printing approaches have consequently introduced spatially varying crosslink densities into monolithic photothermal hydrogel actuators, producing programmable bending while preserving structural continuity. Heterogeneous arrangements of PNIPAM and PNIPAM/rGO regions can additionally generate self-shadowing and secondary illumination, enabling oscillation or multistage deformation under a stationary light source [43].

Photothermal energy can also be converted into motion without relying on conventional LCST-mediated deswelling. In Figure 2D, iron oxide nanoparticles embedded in an elastic poly(sodium acrylate) hydrogel rapidly raise the local temperature above the boiling point of water. Vaporization produces a transient bubble-like protrusion, and its expansion against the substrate generates an impulsive reaction force. The mechanism enables rapid in-air jumping and rolling that are substantially faster than diffusion-limited swelling actuation [18]. This example establishes an important distinction between quasi-static photothermal morphing and impulsive photothermal propulsion: the former converts a thermodynamic volume transition into sustained curvature, whereas the latter temporarily stores energy in a pressurized vapor cavity and releases it over a short interval.

Accordingly, the performance of a photothermal hydrogel robot cannot be evaluated solely from its maximum temperature or bending angle. Relevant parameters include optical power density, wavelength, heated volume, response and recovery times, mechanical work per cycle, water retention, thermal damage threshold, and the stability of the absorber–network interface. Recent studies increasingly combine material heterogeneity, spectral addressability, and feedback generated by self-shadowing to reduce external control requirements [35,43]. These approaches shift photothermal hydrogels from simple light-triggered elements toward systems in which the geometry and material distribution participate in regulating the actuation sequence.

### 2.2. Photochemical Regulation and Spatial Patterning of Chemical Gels

This section focuses on mechanisms that establish a mechanical state through a light-induced chemical transformation rather than through bulk heating. Photoisomerization, bond cleavage or formation, ligand dissociation, and light-gated redox reactions modify network hydrophilicity, charge, connectivity, or molecular recognition, thereby changing the osmotic and elastic contributions to swelling. Because these reactions can be confined to exposed regions, projected light also functions as a patterning tool for encoding local strain and mechanical heterogeneity [32,44,45].

**Figure 3:**
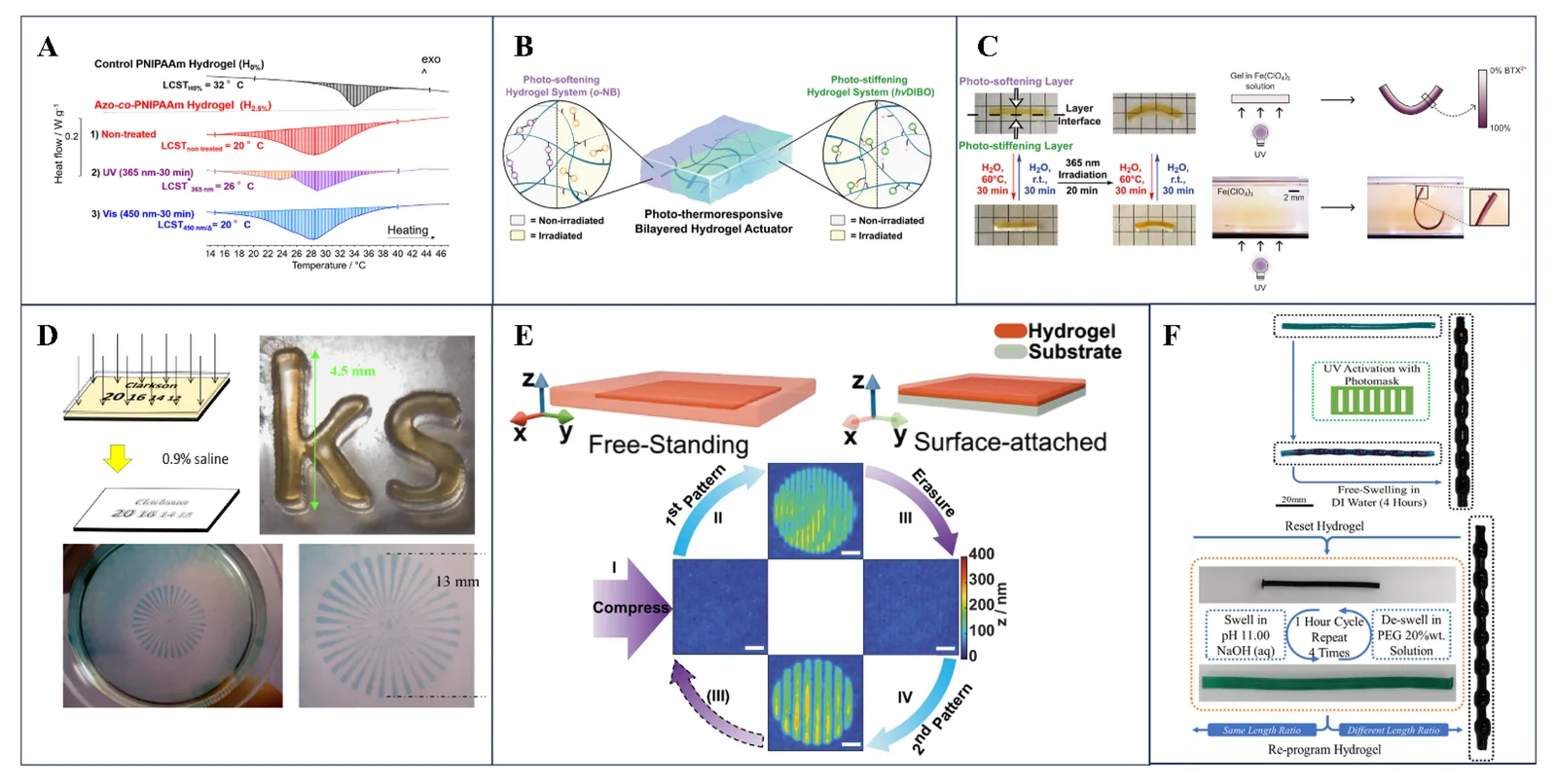
Photochemical regulation and spatial patterning in light-responsive hydrogels. (**A**) Reversible optical modulation of the LCST of azobenzene-functionalized PNIPAM hydrogels [22]. (**B**) Photochemical softening and stiffening used to create patterned mechanical properties and switchable bilayer bending [49]. (**C**) Light-gated oxidation of a redox-active hydrogel, producing localized charge generation, swelling, and bending [24]. (**D**) Mask-assisted photochemical degradation and patterning of Fe(III)-crosslinked alginate hydrogels [25]. (**E**) Reconfigurable optical shaping of free-standing and surface-attached photoresponsive hydrogel films [53]. (**F**) Photomask-defined reprogramming of responsive regions in a hydrogel-based mechanical metamaterial [54]. The panels were adapted and assembled from the cited open-access articles.

Molecular photoswitches enable reversible regulation without permanently severing the network. Azobenzene, for example, undergoes reversible *E*–*Z* isomerization accompanied by changes in molecular geometry, dipole moment, and hydrophobicity. When incorporated into a thermoresponsive network, this molecular change modifies polymer–solvent interactions and can shift the LCST. Figure 3A shows that a relatively low content of an azobenzene comonomer markedly lowers the LCST of PNIPAM, while ultraviolet and visible irradiation reversibly alter the thermal transition through changes in the azobenzene isomer population [22]. Light therefore does not necessarily produce deformation directly; it may instead redefine the temperature range in which a phase transition becomes accessible. This coupling between photoisomerization and thermoresponsiveness allows optical history, environmental temperature, and network composition to jointly determine the actuator state.

Spiropyran-containing hydrogels provide a complementary molecular route. Conversion between spiropyran and merocyanine states changes molecular charge and water affinity, thereby inducing contraction or expansion. Depending on the substitution pattern and network composition, irradiation can either expel water or promote water uptake [46]. Covalent–supramolecular hybrid networks have been used to amplify this molecular event by coupling spiropyran switching to the reorganization of supramolecular domains, producing mechanically reinforced materials capable of bending, walking, and rotation [47]. A related spiropyran hydrogel walker converted cyclic swelling and contraction into directional displacement through asymmetric contact with a ratcheted substrate [48]. These studies show that molecular switching supplies a reversible strain source, but directional robotic motion still requires structural or interfacial rectification.

Photochemistry can also modify the network itself. Figure 3B compares photo-stiffening and photo-softening hydrogel layers. Light-triggered crosslink formation increases local modulus and suppresses swelling, whereas cleavage of photolabile linkers lowers network connectivity and increases local compliance. Combining these reactions in a bilayer enables the bending state to be encoded after fabrication, rather than being fixed entirely during moulding or printing [49]. The underlying concept derives from orthogonal photoconjugation and photocleavage chemistries that permit independent control over network composition and mechanical properties [44,45]. In robotic materials, this distinction is important: reversible photoswitching repeatedly changes the state of an existing network, whereas photobond formation or cleavage can rewrite the mechanical architecture of the actuator.

Figure 3C introduces an additional amplification stage through light-gated redox chemistry. A bisthioxanthylidene-containing hydrogel is first converted by light into a molecular configuration that is more readily oxidized. In the presence of a suitable oxidant, only the illuminated regions develop the charged dicationic state, increasing hydration and swelling through electrostatic and Donnan contributions. The resulting redox state is non-volatile and produces a substantially larger change in swelling than photoisomerization alone. Masked irradiation can therefore generate persistent swelling gradients, reversible surface textures, and bending patterns with high spatial selectivity [24]. This light-gated reaction demonstrates how a low-energy optical signal can control a chemically amplified mechanical response.

Photochemical degradation offers a straightforward means of producing permanent or semi-permanent spatial patterns. In Figure 3D, Fe(III)-crosslinked alginate containing lactate undergoes light-induced reduction and loss of ionic crosslinks. Exposure through a photomask selectively erodes illuminated regions, permitting the fabrication of letters, radial patterns, and thickness-controlled hydrogel features [25]. Related dual ionic and covalent crosslinking methods have produced patterned alginate hydrogels with locally different stiffness and swelling behavior [50]. Photocleavable nucleic-acid crosslinkers have further enabled three-dimensional patterning of hydrogel stiffness and biochemical functionality by conventional photolithography or two-photon excitation [51]. Single-photon lithography on pre-stretched hydrogels can reduce the final feature size after elastic recovery and can place chemical functions within mechanically deformable structures [52].

The mechanical outcome of a photochemical pattern is strongly influenced by confinement. A free-standing film can swell approximately in three dimensions, whereas a film covalently attached to a substrate is restricted in-plane and responds predominantly through thickness variation or surface buckling. Figure 3E exploits this difference in azobenzene-containing hydrogel films. Spatially patterned irradiation changes host–guest interactions and creates erasable surface-relief structures; subsequent illumination can rewrite or translate the pattern. Reducing film thickness shortens the solvent-transport distance, allowing sub-second response and repeated shaping at frequencies that are difficult to obtain in bulk hydrogels [53]. Thus, photochemical kinetics and poroelastic transport must be designed together: a rapid molecular reaction does not guarantee rapid actuation if solvent redistribution remains slow.

Projected light can also reconfigure the mechanical properties of larger hydrogel structures. In Figure 3F, photomask exposure produces spatially defined responsive regions in a hydrogel beam. Subsequent swelling and deswelling alter the distribution of mass and stiffness, enabling reprogramming of the dynamic characteristics of a hydrogel-based metamaterial [54]. Digital light processing extends this approach by fabricating three-dimensional hydrogels that retain photodegradable or photoreactive groups after printing. These groups can later be addressed to create channels, alter local mechanics, or modify the printed geometry [55]. Such methods separate structural fabrication from functional programming and make it possible to revise an actuator after its initial manufacture.

A comparison of these actuation routes indicates that no single mechanism is universally optimal for all light-responsive hydrogel robots. Photothermal actuation can employ a wide range of optical absorbers and established thermoresponsive polymer networks, produce comparatively large deformation over a broad spectral range, and avoid the need to introduce complex photochemical groups at every responsive site. It is therefore advantageous when relatively large displacement, higher force output, or straightforward material preparation is required. Its practical response rate, however, is often jointly controlled by heat diffusion and water migration. Increasing optical power can shorten the heating period but may also cause local overheating, unintended activation of neighbouring regions, and progressive water loss during cycling. Molecular photoswitches can regulate network polarity, hydration, and charge with a smaller bulk temperature increase and are therefore attractive for wavelength-selective addressing and high-resolution spatial programming. Their macroscopic deformation may nevertheless be limited by photoswitch concentration, quantum efficiency, photofatigue, and thermal back-relaxation. Photocleavage, photocrosslinking, and light-gated redox reactions can produce larger or more persistent local mechanical differences and are valuable for structural reconfiguration and spatial patterning, but some reactions are irreversible or require ultraviolet irradiation or additional chemical reagents.

On this basis, future designs should select the energy-conversion pathway according to the intended task rather than maximizing photo-induced volume change alone. Repetitive locomotion requires reversibility, short recovery times, and cyclic stability; structural reprogramming requires spatial resolution and state retention; and biomedical operation requires strict control of temperature, optical dose, and potential photochemical by-products. Orthogonal integration of photothermal and photochemical processes may allow rapid actuation and persistent structural programming to be assigned to different optical channels, thereby reducing the trade-off among speed, stability, and reconfigurability.

## 3. Fabrication Strategies for Light-Responsive Hydrogel Actuators

**Figure 4:**
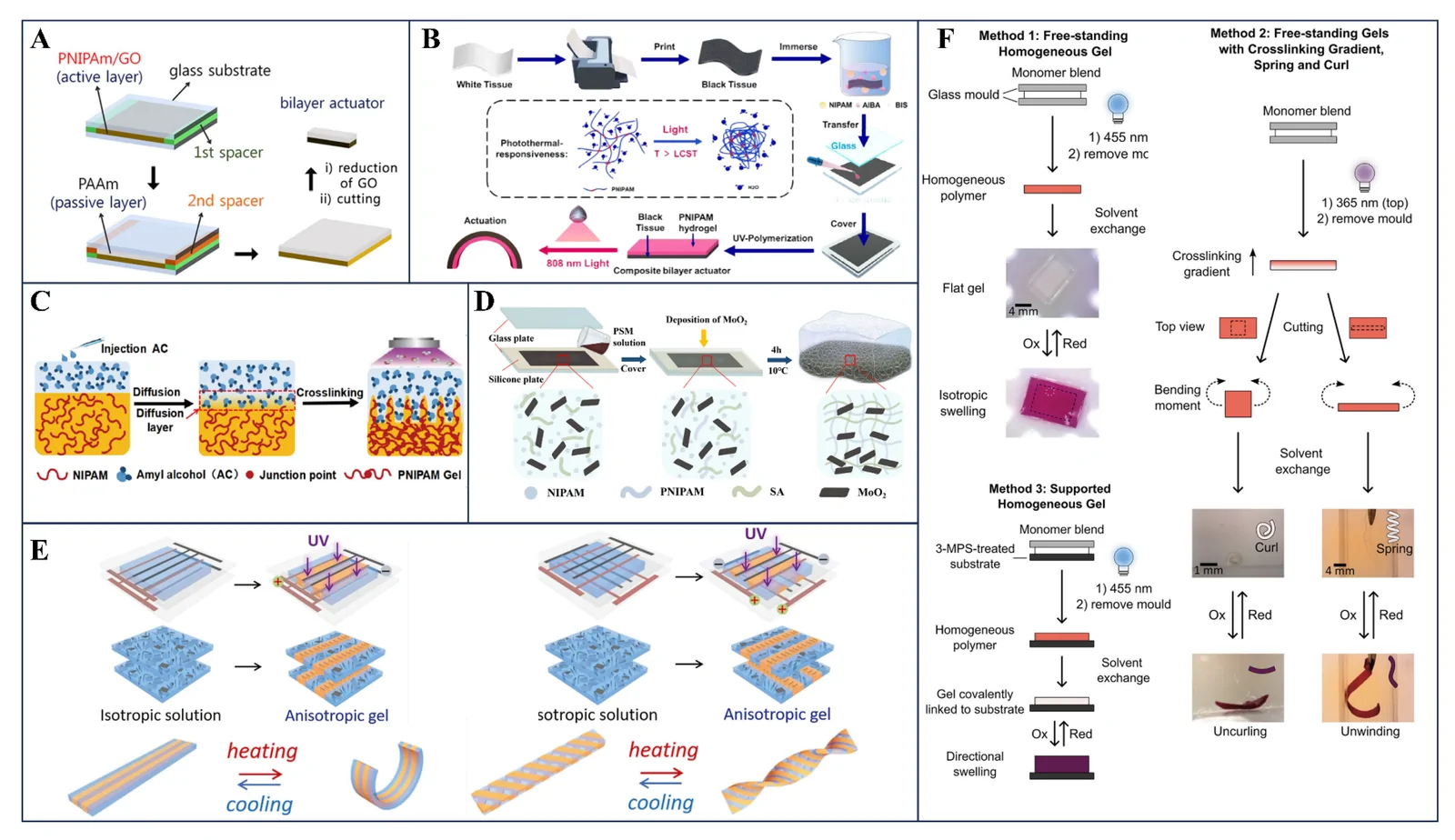
Fabrication of bilayer, gradient, and constrained-polymerization hydrogel actuators. (**A**) Spacer-assisted sequential polymerization of a PNIPAM/graphene-oxide active layer and a PAAm passive layer, followed by graphene-oxide reduction and cutting of the bilayer actuator [40]. (**B**) Inkjet printing of a photothermal pattern on tissue paper, infiltration with PNIPAM precursor, and in situ polymerization to produce a paper/hydrogel composite actuator [56]. (**C**) Liquid stratification and interfacial diffusion used to generate a through-thickness porous gradient in a thermoresponsive hydrogel [57]. (**D**) Gravity-assisted redistribution of MoO_2_ nanosheets during polymerization of a PNIPAM/sodium-alginate composite, producing photothermal and conductive gradients [58]. (**E**) Patterned-electrode-assisted orientation of nanosheets followed by UV polymerization, enabling spatially programmed bending and twisting [59]. (**F**) Fabrication of homogeneous free-standing, crosslink-gradient, and substrate-supported light-gated redox hydrogels via tuning irradiation wavelength and mechanical confinement. [24]. Panels were adapted from the cited open-access articles under their respective Creative Commons licenses.

**Figure 5:**
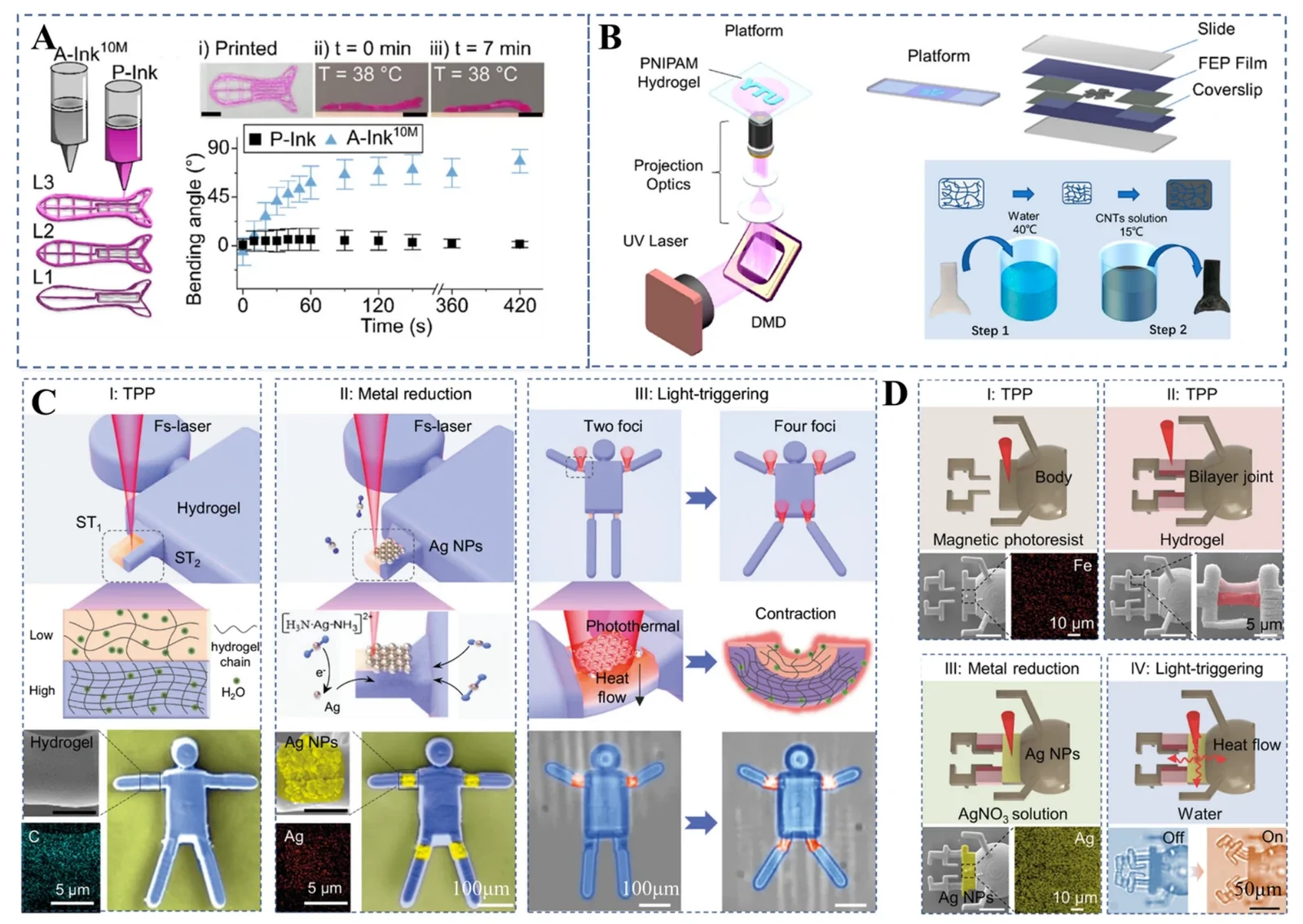
Three-dimensional printing and femtosecond-laser fabrication of light-responsive hydrogel actuators. (**A**) Multi-material extrusion printing of a fish-shaped actuator from active GelMA/PNIPAM and passive GelMA inks; gold nanostars provide near-infrared photothermal activation of the printed tail [62]. (**B**) DMD-based projection curing of PNIPAM microstructures between FEP films, followed by thermally assisted impregnation with carbon nanotubes to obtain photothermal PNIPAM/CNT components [63]. (**C**) Two-in-one femtosecond-laser fabrication combining asymmetric two-photon polymerization of hydrogel joints with selective photoreduction of Ag nanoparticles for localized photothermal actuation [69]. (**D**) Sequential two-photon fabrication of a magnetic microbot body and bilayer hydrogel mandible joints, followed by Ag-nanoparticle photoreduction and light-triggered joint actuation [70]. Panels were adapted from the cited open-access articles under their respective Creative Commons licenses.

Once the actuation pathway has been selected, fabrication determines where active, passive, and mechanically constraining regions are positioned within the robot. Figure 4 and Figure 5 organize the available methods into two complementary groups: bilayer, gradient, and constrained-polymerization routes that encode differential strain through composition or network structure, and additive or laser-based methods that place responsive materials, passive supports, and deformable joints at prescribed three-dimensional locations. These strategies are compared in terms of scalability, spatial resolution, material compatibility, and capacity for post-fabrication programming.

### 3.1. Synthesis and Fabrication of Interfacial and Gradient Composite Hydrogels

The bilayer configuration remains one of the most direct methods for converting the volume-phase transition of a hydrogel into macroscopic bending. An active layer that changes its dimensions under illumination is bonded to a passive or less responsive layer. Because the two layers cannot deform independently, the mismatch strain is released through out-of-plane curvature. The resulting bending direction and magnitude depend on the thickness ratio, elastic-modulus ratio, interfacial adhesion, swelling mismatch, and spatial distribution of the light absorber. Kim et al. constructed a PNIPAM/reduced-graphene-oxide active layer on a passive polyacrylamide layer by spacer-controlled sequential polymerization, followed by reduction of graphene oxide and cutting of the laminated gel (Figure 4A) [40]. Reduced graphene oxide simultaneously served as a broadband photothermal transducer and reinforced the active network. Local irradiation heated the PNIPAM layer above its lower critical solution temperature, whereas the PAAm layer retained its dimensions, producing large and reversible curvature.

The same mismatch principle can be implemented using a porous fibrous substrate rather than two separately cast hydrogel networks. Wu et al. first deposited black photothermal patterns onto tissue paper by inkjet printing and subsequently polymerized PNIPAM within the paper scaffold (Figure 4B) [56]. The fibrous paper restricted in-plane contraction and improved structural integrity, while its printed black regions converted incident light into heat. This in situ infiltration route reduces the risk of delamination because the polymer network develops around and within the paper fibres instead of being joined to the passive layer after polymerization. Related control over photothermal asymmetry was demonstrated by Lee et al., who introduced polydopamine nanoparticles into the active PNIPAM layer of an agarose/alginate–PNIPAM bilayer through repeated oxidative-polymerization cycles [60]. Adjusting the polydopamine loading changed the local photothermal efficiency without altering the overall geometry, enabling different sectors of one actuator to reach different curvatures under identical illumination. These studies show that a bilayer can be programmed through either mechanical asymmetry, optical asymmetry, or a combination of both.

Although bilayers are straightforward to design, their long-term performance is often limited by interfacial stress concentration, differential fatigue, and hydrolytic weakening of the bonded interface. Monolithic gradient hydrogels alleviate some of these problems by distributing the mismatch continuously across the thickness. Dong et al. used liquid stratification and interfacial diffusion to generate a heterogeneous porous PNIPAM network (Figure 4C) [57]. When amyl alcohol contacted the aqueous precursor, solvent exchange and diffusion produced a graded porous layer before crosslinking was completed. The pore-size gradient shortened the solvent-transport pathway and created an asymmetric deswelling rate, allowing the initially homogeneous precursor to acquire directional thermoresponsive deformation. Because functional particles can be introduced through the diffusional liquid, this method also provides a route for incorporating photothermal agents without preparing a separate active layer.

A different gradient-forming mechanism was used by Liu et al. for the PNIPAM/sodium-alginate/MoO_2_ hydrogel shown in Figure 4D [58]. During in situ polymerization, the MoO_2_ nanosheets sedimented under gravity and interacted differently with the hydrophilic precursor components. This process generated a through-thickness gradient in MoO_2_ content, network morphology, photothermal conversion, and electrical conductivity. Under near-infrared irradiation, the MoO_2_-rich region heated and contracted more strongly than the opposite surface, producing bending without a discrete bonded interface. The conductive gradient additionally allowed resistance changes to be correlated with deformation, integrating actuation and proprioceptive sensing within the same gel. From a fabrication perspective, sedimentation is attractive because it transforms an otherwise undesirable instability of particle-filled precursors into a useful structural asymmetry. Its reproducibility, however, depends on controlling nanosheet concentration, viscosity, polymerization time, mold orientation, and the relative rates of settling and gelation.

Anisotropy can also be generated by orienting reinforcing elements before the network is fixed. Zhu et al. placed patterned electrodes on the confining substrates and used the resulting spatial electric fields to orient charged nanosheets within a hydrogel precursor (Figure 4E) [59]. Ultraviolet polymerization subsequently immobilized the locally defined orientation. Because the direction of nanosheet alignment governed the anisotropic swelling and stiffness of each region, straight, oblique, and orthogonally crossed electrode patterns could encode bending, twisting, and more complex coupled deformations in a single gel sheet. A complementary structural route was reported by Gregg et al., who polymerized PNIPAM directly around photolithographically patterned, vertically aligned carbon-nanotube forests [61]. The nanotube scaffold provided optical absorption, rapid heat transfer, mechanical reinforcement, and a fixed anisotropic architecture. Consequently, the CNT/PNIPAM microactuators converted isotropic network deswelling into rapid lattice contraction, pore closure, local rotation, and optical switching.

Confinement during photopolymerization offers another means of encoding asymmetry without changing the nominal precursor composition. Weber et al. compared three fabrication geometries for a light-gated redox-responsive hydrogel containing a polymerizable benzothioxanthene switch (Figure 4F) [24]. Illumination at a weakly absorbed wavelength produced a nearly homogeneous free-standing gel. In contrast, irradiation at an absorbed wavelength generated a depth-dependent conversion and crosslink-density gradient, from which curling or spring-like structures could be cut. Polymerization on a silane-treated substrate produced a covalently attached hydrogel whose in-plane expansion was constrained, converting redox-induced swelling into directional surface relief. Thus, the same monomer mixture yielded flat, curled, or supported structures by altering only the irradiation wavelength and boundary conditions. This example emphasizes that optical attenuation, often regarded as a processing limitation, can serve as a design parameter for encoding through-thickness mechanical gradients.

Collectively, the methods in Figure 4 illustrate three levels of structural programming. Discrete bilayers amplify the mismatch between two mechanically distinct components; monolithic gradients distribute the mismatch and reduce abrupt interfacial stresses; and field- or light-constrained polymerization defines local anisotropy within a nominally single material. Bilayers are generally the easiest to scale and characterize, whereas continuous gradients offer improved interfacial durability. Field-assisted and patterned-photopolymerization approaches provide greater spatial programmability but require accurate control of electric fields, optical dose, precursor absorption, and polymerization kinetics. Selection among these routes should therefore be based not only on the maximum achievable deformation, but also on cyclic stability, manufacturing tolerance, interfacial integrity, and compatibility with the intended optical stimulus.

### 3.2. Advanced Micro/Nano Manufacturing of Photoresponsive Gels

Additive manufacturing extends hydrogel fabrication from simple sheets and beams to architectures in which geometry, composition, infill direction, and local crosslink density can be independently specified. For light-responsive robots, an effective printing process must satisfy two optical requirements: the precursor must polymerize under the fabrication wavelength, while the resulting structure must absorb or respond to the actuation wavelength without undergoing unintended curing or degradation. Direct-ink writing and projection lithography are suitable for millimetre-scale robots, whereas two-photon polymerization provides submicrometre-to-micrometre resolution for articulated microactuators.

Pendlmayr et al. demonstrated a multi-material extrusion-printing strategy using passive GelMA-based ink and active GelMA/PNIPAM-based ink (Figure 5A) [62]. The distribution of active and passive filaments across three printed layers determined the bending direction and magnitude. Incorporating the asymmetric laminate into the tail of a fish-shaped actuator translated the material-level swelling mismatch into an anatomically localized motion. Gold nanostars were included as plasmonic photothermal transducers, permitting near-infrared irradiation to heat the printed structure and repeatedly bend the tail. This design illustrates an important advantage of extrusion printing: the printing path determines not only the outer shape but also the internal arrangement of responsive and non-responsive filaments. Nevertheless, nozzle diameter, filament continuity, layer-to-layer bonding, ink rheology, and post-printing photocrosslinking remain important constraints on resolution and repeatability.

Wang et al. used a digital-micromirror-device projection system to fabricate patterned PNIPAM structures between fluorinated ethylene propylene films (Figure 5B) [63]. Projection curing simultaneously solidified an entire two-dimensional pattern, providing higher throughput than point-by-point scanning. Because carbon nanotubes strongly absorb the curing light and can interfere with accurate photopolymerization, the authors separated shape formation from functionalization. A pure PNIPAM structure was printed first, collapsed in water above its lower critical solution temperature, and then transferred into a cold CNT dispersion. Reswelling drew the nanotubes into the porous network, and repeated thermal cycling removed weakly bound material. This post-loading strategy preserved print resolution while endowing the structure with photothermal responsivity. The resulting PNIPAM/CNT components were integrated into a swimming robot, flexible arm, and micromanipulator, showing how projection printing can couple rapid patterning with subsequent functional-particle incorporation.

Recent printed hydrogel systems have further refined the relationship among toolpath, internal architecture, and optical actuation. Nishiguchi et al. used multiphoton lithography to print PNIPAM–gold-nanorod nanocomposites with locally varied exposure density, thereby creating active and relatively inactive regions from a single formulation [64]. The programmed voxel density controlled swelling, stiffness, and photothermally induced deformation at submicrometre resolution. At a larger scale, Ghelardini et al. directly printed PNIPAM-based inks containing bovine-serum-albumin-functionalized gold nanorods within a removable gelatin support bath [65]. The support matrix prevented spreading of the low-viscosity ink during printing and photocrosslinking, while the retained gold nanorods enabled reversible photothermal contraction. Li et al. employed extrusion-induced alignment of short carbon fibres in a PNIPAM composite ink to fabricate anisotropic, conductive, and self-sensing hydrogel actuators [66]. These examples demonstrate that additive manufacturing can encode deformation through material placement, exposure dose, filament orientation, or particle alignment rather than relying exclusively on a macroscopic bilayer.

Femtosecond-laser writing provides a further increase in spatial precision because two-photon absorption restricts polymerization to the focal volume. Hippler et al. showed that local exposure dose during three-dimensional laser lithography could regulate the crosslink density and thermomechanical properties of PNIPAM microstructures [67]. Regions written at different doses behaved as microscale bimaterial elements even though they originated from one photoresist. Focused femtosecond irradiation could subsequently produce localized photothermal heating, allowing individual structures to bend without globally changing the temperature of the surrounding liquid. Zheng et al. similarly fabricated PNIPAM–gold-nanorod microactuators by two-photon polymerization and programmed their deformation by varying the printed density and thickness of adjacent regions [68]. These studies established exposure dose as a digital manufacturing variable that can replace the physical assembly of separate active and passive materials.

The multi-joint microactuator in Figure 5C was fabricated by a two-in-one femtosecond-laser process developed by Xin et al. [69]. First, asymmetric two-photon polymerization generated hydrogel joints containing regions with different crosslink densities. The same laser platform was then used to photoreduce silver ions selectively on the hydrogel surface, forming densely distributed Ag nanoparticles at the intended joints. These nanoparticles acted as local photothermal transducers. Because the rigid body, compliant joint, crosslink-density asymmetry, and light absorber were defined with micrometre-scale precision, individual joints could be addressed by structured light and actuated within tens of milliseconds. The approach avoids manual assembly and registration errors between separately fabricated components, although the sequential writing and reduction steps limit manufacturing throughput.

Ren et al. extended this method to the ant-inspired microbot shown in Figure 5D [70]. A magnetic photoresist was first written into a rigid body by two-photon polymerization. Bilayer hydrogel joints with unequal laser exposure doses were then integrated at the mandibles, followed by femtosecond-laser reduction of Ag nanoparticles. The magnetic body supplied translation and orientation, whereas the light-responsive joints provided rapid opening and closing of the mandibles. This multimaterial sequence spatially separated locomotion, deformation, and photothermal conversion while maintaining mechanical continuity at the microscale. It also demonstrates that femtosecond processing can integrate materials that would be difficult to combine in a single printable precursor.

The evolution of laser-based manufacturing is also moving from thermally mediated actuation toward direct molecular photoswitching. MacKay et al. recently used stereolithographic printing to prepare methacrylate hydrogel lattices containing azobenzene actuator units [71]. Ultraviolet–visible irradiation induced a volumetric contraction of the printed lattices and enabled pulsatile payload release. Although the demonstrated structures were not complete mobile robots, the study is relevant to future hydrogel machines because it integrates three-dimensional fabrication and molecular photoactuation within one covalent network, thereby eliminating the need for an added photothermal filler.

The advantages of different fabrication methods depend on robot scale and intended function and cannot be ranked solely according to manufacturing resolution. Sequential casting, interfacial polymerization, and sedimentation-based gradient formation require relatively simple equipment, are readily applicable to millimetre- and centimetre-scale actuators, and can provide comparatively high force through a large effective cross-sectional area. These methods are therefore suitable for bending strips, grippers, and larger swimming structures. However, bonded bilayer interfaces may undergo fatigue or delamination, while sedimentation- and diffusion-generated gradients are sensitive to precursor viscosity, gelation time, and mould orientation. Extrusion printing directly regulates material composition, filament orientation, and internal toolpaths and is suitable for producing mechanically continuous multimaterial robots, but nozzle diameter, ink rheology, and interlayer adhesion limit the smallest achievable feature size. Digital light processing provides high planar throughput and reproducible patterning, although absorbing fillers can interfere with curing-light propagation and create a conflict between printing fidelity and subsequent photothermal efficiency. Two-photon polymerization and femtosecond-laser processing provide precise control over microscale joints, crosslink density, and metal-nanoparticle placement and are therefore valuable for multi-degree-of-freedom micromachines. Their limitations include restricted writing volumes, long serial-processing times, and sensitivity to precursor absorption and particle scattering.

In our view, the most practical route is not to manufacture an entire robot using a single high-resolution process, but to adopt hierarchical fabrication. High-throughput printing can form the body and load-bearing structures, whereas localized laser processing can define joints, absorbing regions, and sensing elements. Such a strategy offers a more practical balance among manufacturing efficiency, local precision, material compatibility, and functional integration. Future studies should also report printing resolution, manufacturing time, dimensional error, structural shrinkage, interfacial strength, and cycle-to-cycle consistency rather than evaluating a fabrication method only from its minimum feature size.

## 4. Structural Architectures of Light-Responsive Hydrogel Soft Robots

**Figure 6:**
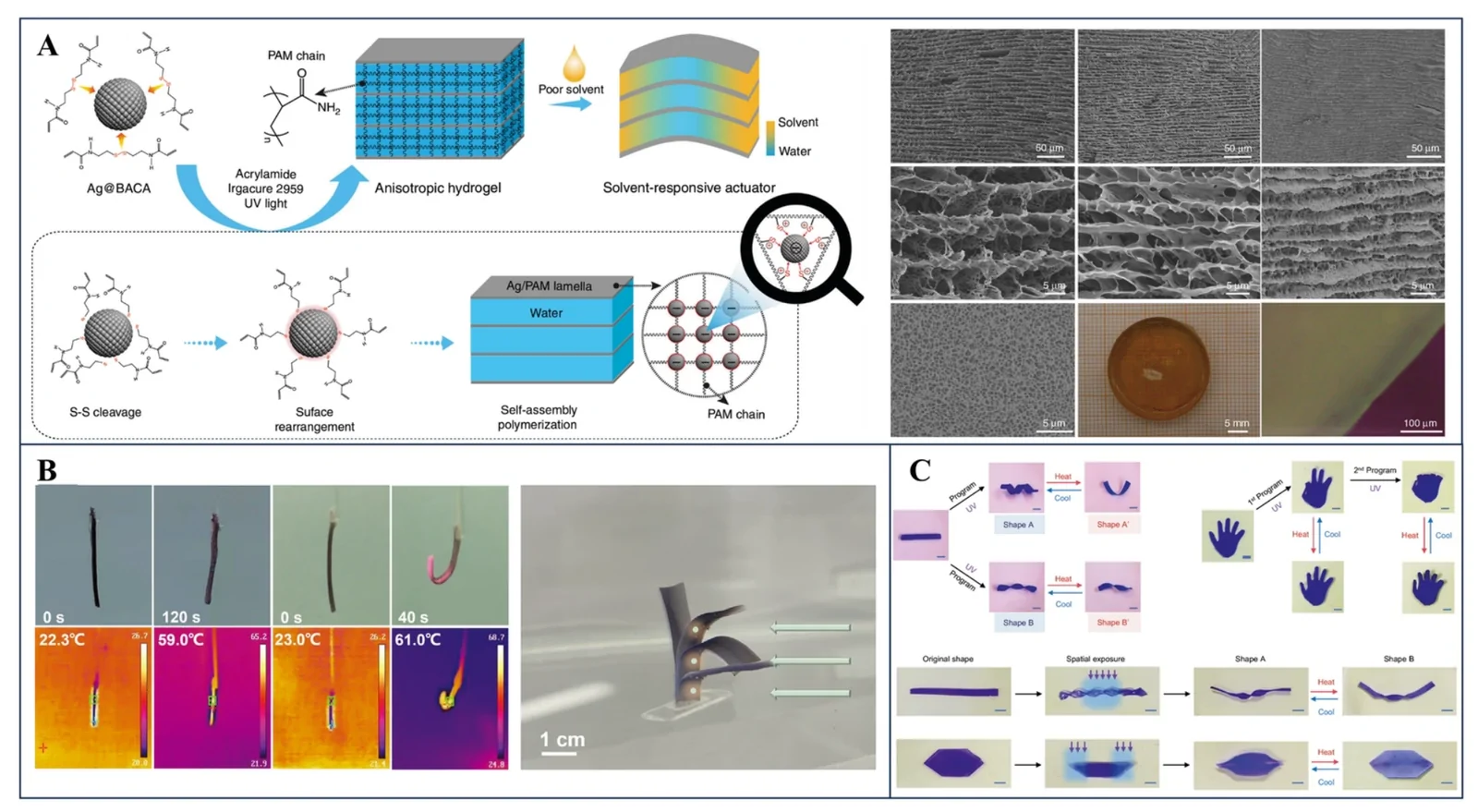
Bilayer, anisotropic, and programmable monolayer architectures of responsive hydrogel actuators. (**A**) Formation of an ordered lamellar Ag/polyacrylamide network through nanostructure self-assembly and in situ polymerization, together with its anisotropic morphology and directional actuation characteristics [73]. (**B**) Photothermal bending of homogeneous and electric-field-programmed gradient Au/g-C_3_N_4_–PNIPAM hydrogel actuators under 808 nm NIR irradiation, together with position-dependent control of the bending deformation [72]. (**C**) Photo-mechanical programming and reprogramming of a dynamically crosslinked PNIPAM monolayer, through which spatially selected regions acquire different reversible deformation modes [16]. Panels were adapted from the cited open-access articles.

**Figure 7:**
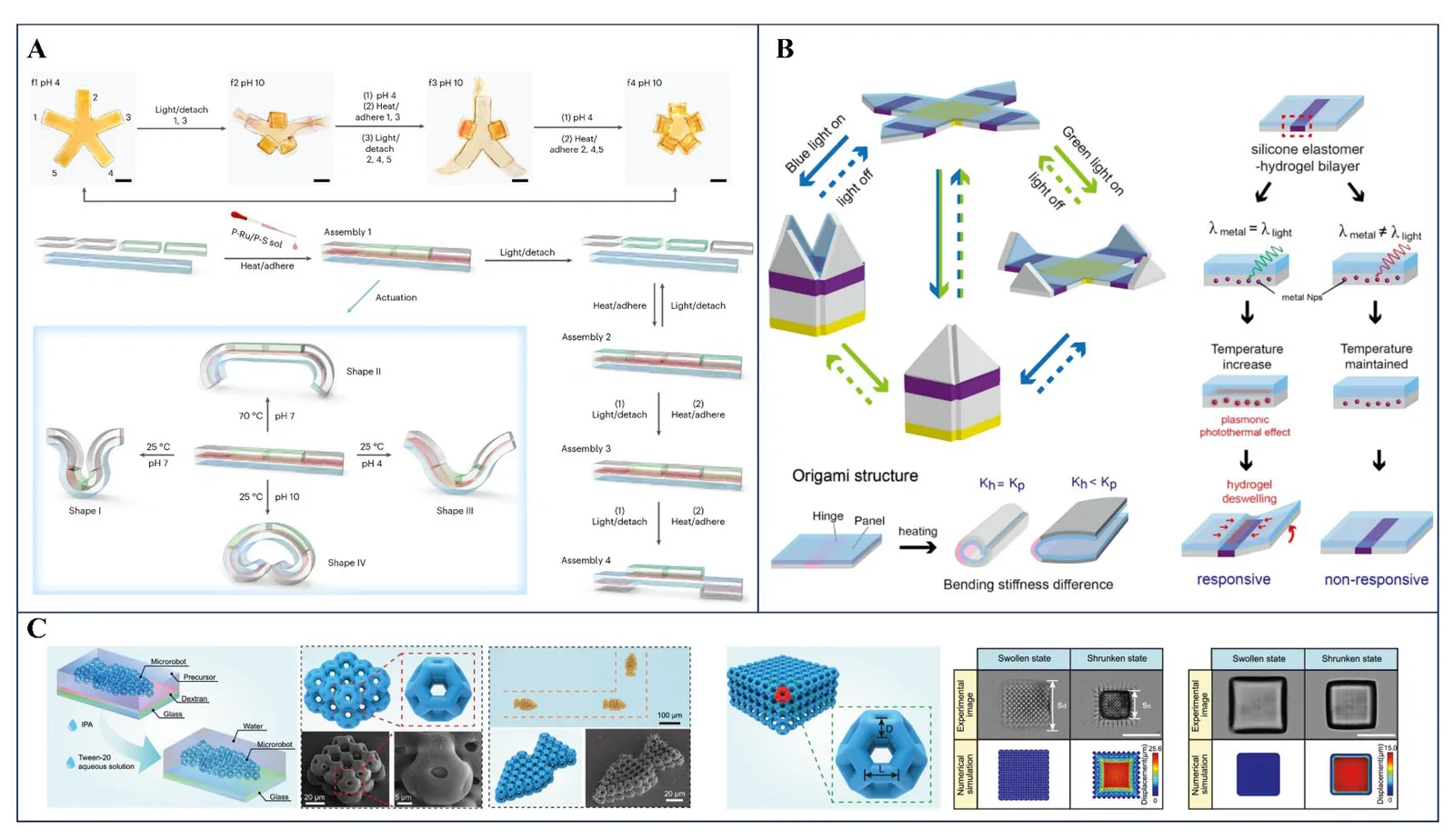
Modular, wavelength-selective, and lattice architectures for reconfigurable hydrogel soft robots. (**A**) Hydrogel units reversibly joined by a Ru–S metallopolymer adhesive, enabling heating-induced adhesion, light-induced detachment, and structural reconfiguration into assemblies with different stimulus-dependent shapes [75], scale bar: 500 μm. (**B**) Wavelength-selective silicone-elastomer/PNIPAM bilayers containing plasmonic metal nanoparticles, in which spectrally addressed hinges and hinge–panel stiffness differences generate multiple origami-like configurations [76]. (**C**) Fabrication and structural characterization of a PNIPAM–SWNT lattice microrobot, including the truncated-octahedron unit cell and deformation of the open lattice during swelling and contraction [80]. Panels A and C were adapted from open-access articles distributed under CC BY 4.0.

The preceding section addressed how spatial heterogeneity is introduced during fabrication; this section examines how that heterogeneity is organized to control strain propagation at the robotic scale. Figure 6 and Figure 7 progress from material-scale architectures—including anisotropic networks, internal gradients, and programmable monolayers—to body-scale architectures such as modular assemblies, wavelength-selective folds, topological structures, and three-dimensional lattices. The comparison therefore focuses on how connectivity, topology, and geometric constraints expand the accessible deformation modes without requiring additional tethered actuators.

### 4.1. Anisotropic and Programmable Monolithic Gel Architectures

Bilayers provide a reference architecture in which strain mismatch is localized at a bonded interface, but the systems considered in this section increasingly encode equivalent mechanical asymmetry within a continuous network. Lamellar alignment, concentration or crosslinking gradients, and reprogrammable chain orientation redistribute swelling strain and solvent transport without necessarily requiring a permanently distinct passive layer. Figure 6 compares these approaches according to whether anisotropy is fixed during synthesis, introduced through an applied field, or rewritten after fabrication.

Figure 6B illustrates an alternative strategy in which directional deformation is encoded within a monolithic gradient hydrogel rather than through the assembly of two compositionally distinct layers. Chen et al. incorporated gold-decorated graphitic carbon nitride nanoparticles (Au/g-C_3_N_4_ NPs) into a thermoresponsive PNIPAM network and applied a direct-current electric field during polymerization. Migration and reorientation of the negatively charged nanosheets generated through-thickness gradients in nanoparticle concentration, pore size, and network density. Under 808 nm near-infrared irradiation, the Au nanoparticles enhanced light absorption and photothermal conversion, driving the PNIPAM network above its lower critical solution temperature and inducing water expulsion and contraction. Although the homogeneous hydrogel and the gradient actuator reached comparable temperatures of approximately 59 and 61 °C, respectively, only the gradient actuator developed pronounced bending because its asymmetric network converted volumetric contraction into a differential strain across the thickness. The bending direction and deformation profile could be further regulated by changing the position of localized illumination, demonstrating that an internally programmed gradient can provide spatially addressable actuation without a discrete passive layer [72].

Bilayers need not contain one active and one inactive component. Li et al. combined two spiropyran-functionalized hydrogels that underwent opposite dimensional changes under the same visible-light stimulus. One layer expanded through a light-induced increase in charge density, whereas the second contracted following photoisomerization and dehydration. Coupling the two active responses increased both the strain difference and the bending rate relative to active–passive combinations. Sequences of these bilayers and light-inactive connectors produced predictable origami-like morphologies and directional crawling [20]. This synergistic configuration is important because it treats both layers as functional components instead of using half of the actuator merely as a mechanical constraint. Locally illuminated spiropyran hydrogel sheets have similarly been designed with photoactive fold lines whose width, orientation, and illumination direction determine the final folding angle [21]. Such designs extend bilayer mechanics toward addressable hydrogel origami.

Internal anisotropy offers an alternative to discrete interfaces. Instead of joining two separately prepared layers, the polymer chains, nanosheets, pores, or crosslinking domains are organized within a continuous network. The resulting directional differences in modulus, permeability, and equilibrium swelling can transform nominally isotropic molecular responses into bending, twisting, or uniaxial contraction. Figure 6A presents a Ag/polyacrylamide hydrogel in which thiolate-functionalized silver nanostructures self-assemble into ordered lamellae and simultaneously act as multifunctional crosslinking domains [73]. The aligned lamellae generate pronounced mechanical and swelling anisotropy, while dynamic thiolate–Ag coordination enables healing under near-infrared irradiation or acidic conditions. The macroscopic actuation demonstrated in the original study was primarily induced by solvent exchange rather than light. Therefore, this work should be regarded as an architectural model for integrating anisotropy and light-assisted repair, rather than as evidence of exclusively light-powered locomotion. Nevertheless, the design is instructive for hydrogel robots because damaged segments with different lamellar orientations can be reconnected to form mechanically integrated actuators with more complex deformation modes.

A third strategy is to create spatially programmed anisotropy within a single hydrogel body. This approach avoids the sharp interface of conventional bilayers and allows the same monolithic sheet to be reprogrammed for different tasks. In the system shown in Figure 6C, a PNIPAM network containing dynamic disulfide crosslinks was mechanically deformed and locally exposed to ultraviolet light. Photoactivated disulfide exchange reorganized the network under load, fixing a locally oriented chain configuration. The programmed regions subsequently displayed rapid and reversible thermal deformation, and repeated irradiation allowed the deformation field to be erased or rewritten. Incorporation of carbon-based photothermal fillers converted near-infrared irradiation into localized heating, enabling a single hydrogel robot to be reconfigured between swimming, crawling, and rotational modes [16]. Because the dominant response originated from thermally driven conformational changes of oriented chains rather than long-range water diffusion, the programmed actuator operated considerably faster than conventional bulk PNIPAM gels.

The distinction between bilayer and monolayer architectures is consequently more functional than geometrical. A bilayer stores mechanical incompatibility at a material interface, whereas a programmed monolayer stores it as a spatial variation in network orientation, crosslink density, or residual stress. Optical manufacturing can further blur this distinction. Dynamic light projection has been used to encode spatially varying swelling ratios and response rates in initially planar thermoresponsive hydrogels, producing structures with prescribed Gaussian curvature and sequential shape transitions [74]. In that case, light was principally used to program the network during fabrication, while subsequent actuation was thermal. Such studies nevertheless establish design rules that can be transferred to photothermally operated robots by adding spatially selective absorbers.

Overall, the choice among a bilayer, anisotropic bulk gel, and programmable monolayer should be based on the required mechanical output. Bilayers are advantageous when large and predictable curvature is required; anisotropic networks are better suited to directional contraction, twist, and damage-tolerant operation; and programmable monolayers provide greater freedom to rewrite local actuation axes without reconstructing the robot. Future architectures are likely to combine these strategies—for example, by placing reprogrammable anisotropic domains inside an interfacially robust bilayer—so that amplitude, direction, and sequence can be controlled independently.

### 4.2. Topological Design and Reconfigurable Assemblies in Hydrogel Systems

As the geometric complexity of a hydrogel robot increases, prescribing only the final deformation is insufficient. Individual regions must often deform in a particular order to prevent mechanical interference, establish directional motion, or complete a multistep task. Temporal programming can be introduced through differences in thickness, crosslink density, absorber concentration, optical wavelength, thermal relaxation, or solvent-transport distance. At a still higher level, topology and cellular architecture can regulate how local deformation propagates through the robot. These strategies shift part of the control burden from the external illumination system into the physical organization of the body.

A modular route is presented in Figure 7A. Liu et al. developed a photocontrolled metallopolymer adhesive based on reversible Ru–S coordination and polymer-chain entanglement. Heating promoted coordination and adhesion between hydrogel units, whereas blue-light irradiation dissociated the coordination complex and allowed the units to detach. Temperature- and pH-responsive modules could consequently be assembled, optically disassembled, and recombined into different configurations with distinct shape-change programs [75]. Importantly, light did not directly generate all of the mechanical deformation in these assemblies; rather, it controlled connectivity and therefore redefined how the thermally or chemically active modules interacted. This distinction identifies a broader function of optical stimulation: light can program the topology of a robot even when another stimulus supplies the actuation energy.

Figure 7B demonstrates direct optical selection of different folding pathways. The hybrid robot consists of a PNIPAM hydrogel bonded to silicone-elastomer regions containing plasmonic metal nanoparticles. Spectrally differentiated Ag- and Au-containing regions convert blue or green illumination into localized heating, driving PNIPAM deswelling only in the corresponding hinge regions. Simultaneously, the use of soft hinges and stiffer panels suppresses distributed rolling and concentrates curvature at predefined fold lines. Different combinations of the two illumination channels produce four planar or three-dimensional states, including open, claw-like, box-like, and closed configurations [76]. This study shows that wavelength can operate as an optical address for different structural elements, while the hinge–panel stiffness contrast determines whether local contraction becomes smooth curvature or origami-like folding.

Temporal shells provide a related architectural concept in which the order of deformation is encoded in the body rather than selected through multiple light beams. Guseinov et al. introduced an actuation-time landscape by varying the thickness of softening brackets in a tessellated shell surrounding a prestretched membrane. Different regions released stored elastic energy at different times, allowing a planar structure to reach a prescribed three-dimensional surface without destructive collisions [77]. This particular shell was thermally actuated in warm water and was neither a hydrogel nor a light-responsive robot; it is therefore included here as a mechanical design precedent rather than as a photoresponsive hydrogel example. In hydrogel systems, an analogous time landscape can be generated by spatially varying optical absorption, gel thickness, porosity, crosslink density, or phase-transition temperature. The phototunable swelling kinetics demonstrated in printed thermoresponsive hydrogel sheets provide an experimentally relevant route for transferring this temporal-shell concept to light-programmed soft robots [74].

Topology introduces constraints that cannot be removed by continuous deformation without cutting or reconnecting the body. Zhu et al. exploited this property in hydrogel “knotbots” formed from anisotropic PNIPAM strands containing aligned nanosheets and Au nanoparticles. Trefoil, pentafoil, toroidal, and linked configurations store topology-dependent prestress and exhibit nonuniform illumination because one section of the knot can shadow another. Under uniform or scanned light, the interaction among photothermal contraction, self-contact, stored torsion, and self-shadowing repeatedly breaks and restores symmetry, producing continuous rolling, spinning, and rotation [78]. In contrast to robots controlled by a sequence of externally prescribed light spots, the knot itself participates in the feedback process. Topology therefore functions as a form of embodied regulation: the same material and illumination can yield different behaviors when the strand is tied into a different invariant configuration.

Patterned interfaces can also create reconfigurable interactions between separate hydrogel bodies. Metal-nanoparticle patterns formed inside thermoresponsive hydrogel films generate localized wrinkling under light at an air–water interface. The accompanying distortion of the three-phase contact line produces attractive or repulsive capillary interactions, depending on the illuminated pattern. Consequently, individual “gel surfers” can change shape, assemble into selected configurations, and undergo sustained translation or rotation without permanent mechanical joints [79]. Whereas the metallopolymer adhesive in Figure 7A alters topology through bond formation and dissociation, these interfacial assemblies use light-regulated capillary forces to create transient mechanical connectivity.

Lattice architectures provide another means of amplifying hydrogel deformation while reducing the amount of active material. The light-driven microrobot in Figure 7C was fabricated from a PNIPAM–single-walled carbon nanotube hydrogel with a truncated-octahedron lattice. The open cellular geometry reduces relative density and bending stiffness, shortens heat- and mass-transfer pathways, and allows individual struts to undergo substantial deformation. Sequential laser scanning can therefore produce localized contraction waves that support peristalsis, in situ rotation, and hopping [80]. Under comparable conditions, the lattice robot required approximately one-sixth of the laser energy and attained about three times the motion speed of a solid hydrogel robot. These results indicate that the lattice is not simply a lightweight version of the bulk gel; its connectivity and local compliance fundamentally alter energy transmission and deformation kinetics.

Related micro-metastructures combine a hydrogel artificial muscle with printed beams whose buckling behavior varies from pixel to pixel. By controlling the printing power, orientation, and geometry of these beams, a common starting pattern can transform into a broad family of thermally selected configurations with different chirality and optical anisotropy [81]. Although actuation in that study was thermal rather than directly optical, the collaborative-buckling principle is relevant to photothermal hydrogel lattices because a local temperature field can be used to select among mechanically encoded modes. More recently, digital light processing combined with complementary dark polymerization has enabled the fabrication of photoresponsive hydrogels in complex gyroid and knotted-lattice geometries while preserving groups for post-print photostiffening, photosoftening, or photodegradation [55]. These structures were demonstrated primarily as programmable materials rather than autonomous robots, but they provide a manufacturing route for future lattices whose geometry and mechanical properties can both be rewritten by light.

Bilayers, continuous gradients, topological structures, and lattices address mechanical conversion at different hierarchical levels. Bilayers use a clearly defined strain mismatch to generate large and predictable curvature and are comparatively straightforward to design and model, but interfacial fatigue and unequal solvent transport may limit long-term cycling. Continuous gradients and anisotropic monolithic gels avoid abrupt material interfaces and can encode the response direction within the network, although their reproducibility and local structural characterization are more demanding. Topological knots and closed ribbons exploit self-contact, prestress, and self-shadowing to establish internal feedback without externally imposed temporal modulation, making them attractive for sustained rolling or rotation. Their behaviour, however, is highly sensitive to initial topology, contact state, and boundary conditions. Open lattices reduce inactive material, shorten heat- and solvent-transport distances, and accelerate response, but a lower relative density may also reduce force output, load-bearing capacity, and defect tolerance. Structural lightweighting therefore does not necessarily improve overall robotic performance.

In our view, future architectures should be designed backwards from the intended task. Rapid microscale locomotion requires short transport distances, payload manipulation requires sufficient effective cross-sectional area and structural stiffness, and autonomous periodic motion requires self-shadowing, contact, or geometric instability to function as a predictable feedback element. Material composition, internal anisotropy, and macroscopic topology should therefore be treated as coupled design variables rather than independent optimization targets. Integrating wavelength-selective hinges, local gradients, and topological constraints within one structure may reduce the number of external control channels and improve autonomous operation.

## 5. Locomotion Modes of Light-Responsive Hydrogel Soft Robots

**Figure 8:**
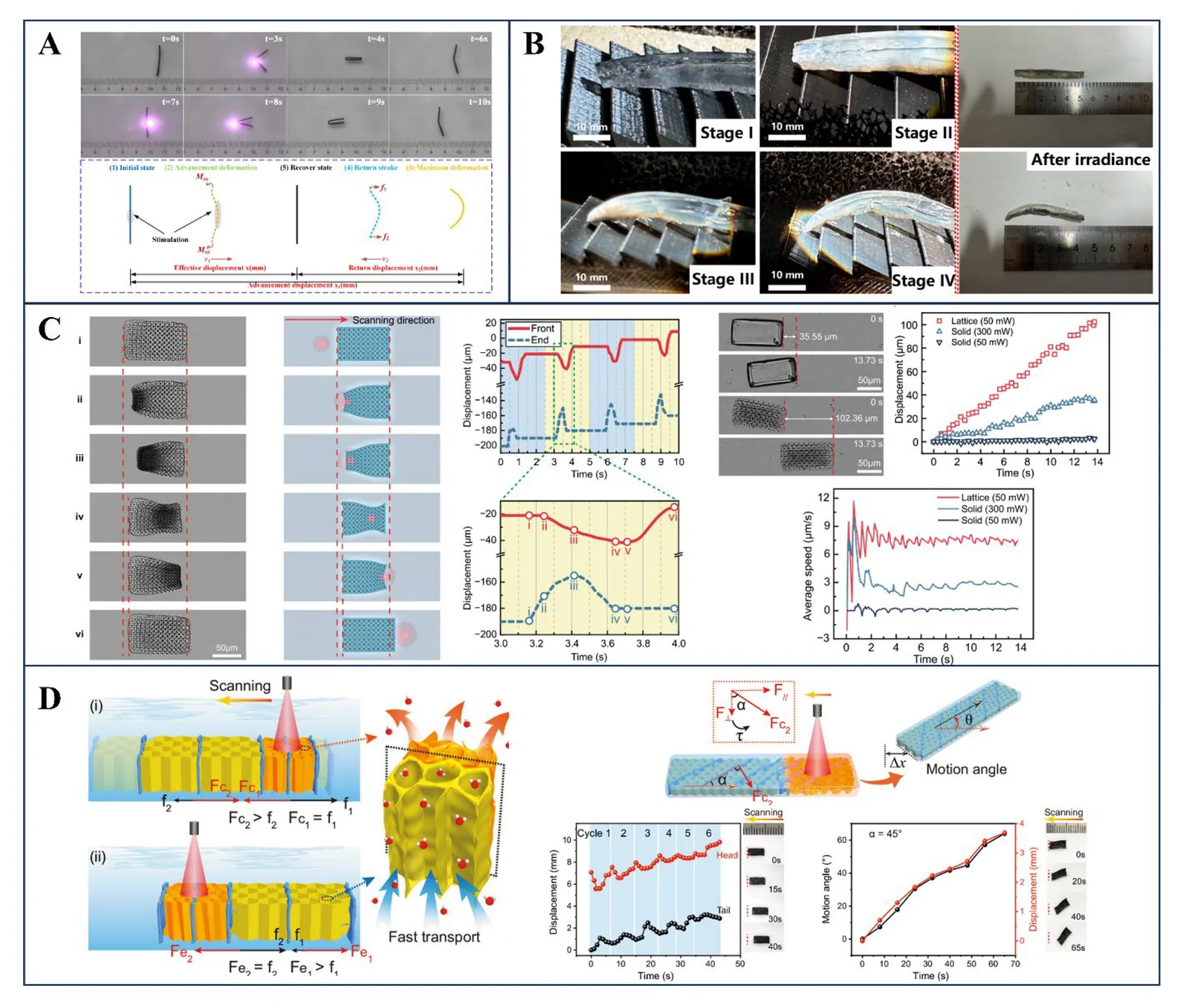
Light-driven crawling, peristaltic locomotion, and friction regulation in hydrogel soft robots. (**A**) Asymmetric bending and recovery of a locally GO-infiltrated PNIPAm–4HBA hydrogel strip produce cumulative forward displacement [82]. (**B**) Ratchet-assisted locomotion of an anisotropic Fe_3_O_4_-containing PNIPAm-based bilayer robot; photothermal bending engages the ratchet substrate, whereas cooling and rehydration restore the body [83]. (**C**) Sequential laser scanning of a PNIPAM–SWNT lattice microrobot generates local shrinkage, reswelling, and friction redistribution for linear peristalsis [80]. (**D**) Scanning-light-driven crawling and steering of a lamellar PNIPAM/CNT hydrogel through the coordination of anisotropic contractile forces and state-dependent friction [84].

**Figure 9:**
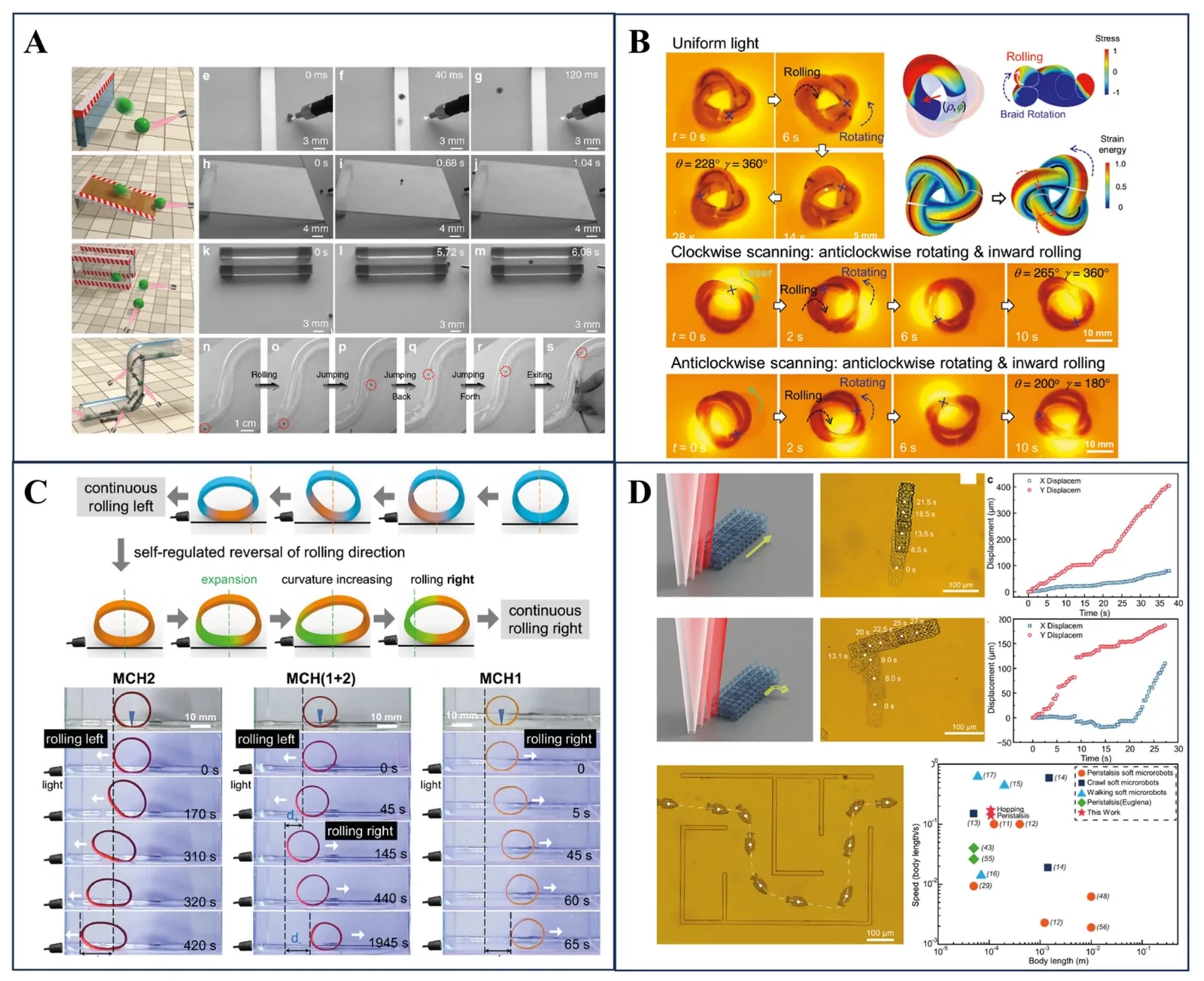
Jumping, rolling, rotation, and continuous hopping of light-responsive hydrogel robots. (**A**) Bubble-mediated jumping, rolling, and obstacle crossing by a photothermal nanoparticle-containing hydrogel actuator [18]. (**B**) Topology- and self-shadowing-regulated rolling and rotation of hydrogel knotbots under illumination [78]. (**C**) Self-regulated forward and reverse rolling of a structurally homogeneous dual-spiropyran hydrogel under constant illumination [87]. (**D**) High-power laser-induced continuous hopping and directional steering of a PNIPAM–SWNT lattice microrobot [80].

**Figure 10:**
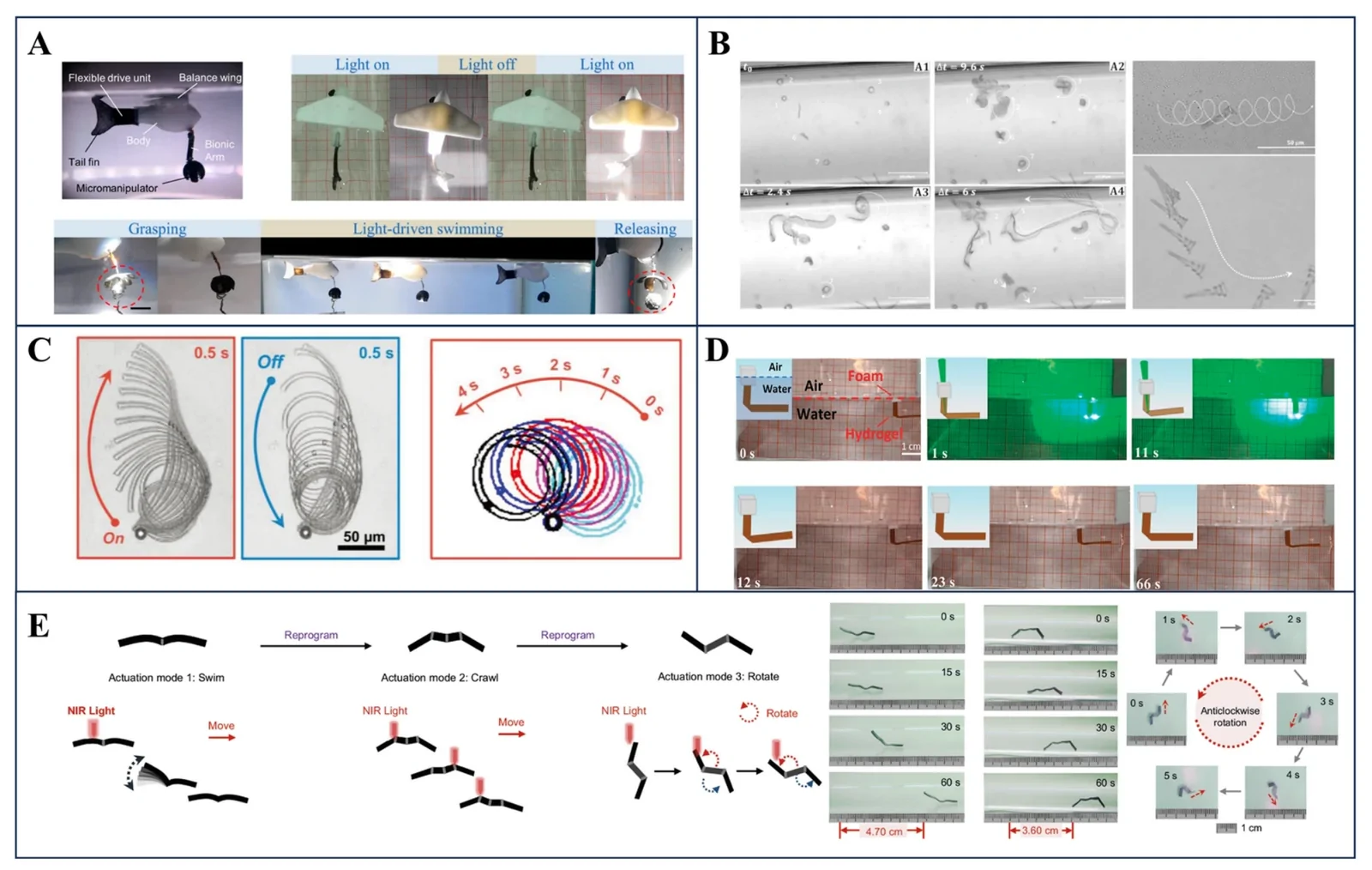
Biomimetic swimming, pulsed microswimming, and underwater locomotion of light-responsive hydrogel robots. (**A**) A multifunctional fish-like robot integrating a PNIPAM/CNT tail actuator with light-controlled swimming, grasping, cargo transport, and release [63]. (**B**) Pulsed-NIR propulsion of tapered PNIPAM/gold bilayer microhelices through non-equilibrium coiling and uncoiling cycles [91]. (**C**) Light-driven rotation of a hydrogel–gold microhelix around a microsphere pivot [92]. (**D**) A green-light-responsive P(NIPAM-co-AM)/Fe_3_O_4_ hydrogel actuator propelling a floating foam platform [93]. (**E**) Photo-mechanically programmed and reprogrammable underwater locomotion—including swimming, crawling, and rotation—in a disulfide-crosslinked PNIPAM hydrogel [16].

**Figure 11:**
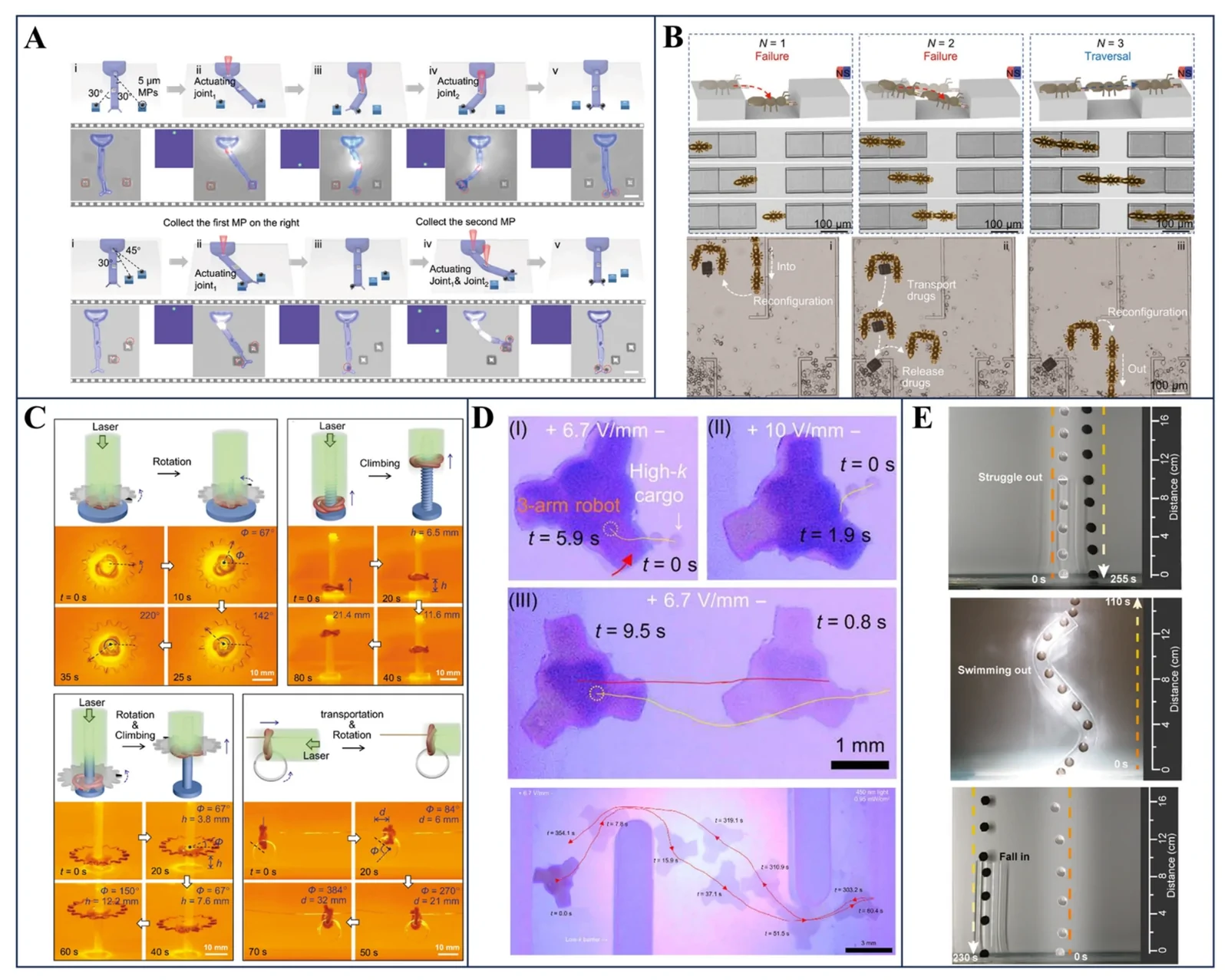
Multimodal locomotion, reconfiguration, and task execution by light-responsive hydrogel robotic systems. (**A**) Selective optical control of a femtosecond-laser-fabricated multi-joint hydrogel microarm for collecting microparticles at different positions [69]. (**B**) Light-controlled mandible actuation and magnetically driven locomotion of reconfigurable ant-inspired microbot collectives [70]. (**C**) Climbing, gear rotation, and object transport by topology-regulated hydrogel knotbots [78]. (**D**) Light-programmed charge reversal coupled with electric-field-driven electrophoretic locomotion, cargo handling, and obstacle avoidance [95]. (**E**) Multimodal underwater navigation of a photo-mechanically inactive rGO/polyacrylamide tough-gel robot through photo-thermo-fluidic coupling [96].

Having established how material and body architectures generate deformation, the next question is how that deformation produces net motion. Figure 8, Figure 9, Figure 10 and Figure 11 are therefore organized according to the mechanism that transfers momentum to the environment: rectified contact and friction for crawling, impulsive deformation or self-shadowing feedback for jumping and rolling, non-reciprocal cycles and fluid coupling for underwater motion, and auxiliary fields for multimodal task execution. This organization distinguishes the observed gait from the physical process that sustains it.

More specifically, the conversion from photoinduced hydrogel deformation to robotic motion can be understood as a multistage mechanical pathway: the incident wavelength and optical dose first alter the local thermal or chemical state of the network; this change generates a spatially non-uniform and time-dependent strain field; the robot architecture redistributes that strain into bending, twisting, contraction, buckling, or elastic-energy storage; and the resulting body deformation produces external contact forces or hydrodynamic stresses. Net locomotion therefore depends not only on the magnitude of swelling, deswelling, or curvature, but also on the location of the active region, the rate and sequence of deformation, the relaxation behavior of the network, and the manner in which the body exchanges momentum with its surroundings. On a solid substrate, photothermal deswelling or photochemical swelling changes the body curvature, normal load, contact position, and interfacial friction. A crawler advances only when the actuation and recovery strokes experience unequal anchoring conditions or follow different mechanical paths; ratchet substrates, spatially scanned illumination, geometric polarity, and state-dependent friction consequently rectify cyclic deformation into a finite step.

Jumping systems concentrate deformation or stored elastic energy into a short release process that generates an impulse, whereas rolling systems continuously relocate their curvature, contact line, and center of mass. In a liquid environment, reciprocal expansion and contraction generally produce limited net translation, particularly at small scales. Effective propulsion instead requires a non-reciprocal deformation cycle, a traveling bending wave, a phase difference between adjacent active regions, an asymmetric propulsive appendage, or a light-induced flow field. Topological knots and porous lattices further redistribute local strain through structural connectivity, enabling deformation of individual segments to produce whole-body rolling, peristalsis, rotation, or hopping.

In hybrid systems, light may primarily regulate joint configuration, surface adhesion, charge state, or local fluid motion, while magnetic, electric, gravitational, or hydrodynamic forces supply part of the translational momentum; these systems should therefore be distinguished from robots whose motion is generated directly by photoinduced network deformation. This deformation-to-motion framework also explains why a large bending angle does not necessarily correspond to high locomotion speed or efficiency, whereas a relatively small but rapidly sequenced local strain may generate substantial displacement when it is effectively rectified. Accordingly, hydrogel deformation and robotic locomotion should be evaluated as a connected process by relating optical input, local strain or curvature, response and recovery rates, contact or fluid forces, displacement per cycle, body-length-normalized velocity, and the contribution of any auxiliary field. Within this framework, Figure 8, Figure 9, Figure 10 and Figure 11 illustrate different strategies for converting internally generated hydrogel strain into externally observable motion and task execution.

### 5.1. Interfacial Friction Regulation and Crawling Locomotion of Gel Actuators

Crawling is among the most direct locomotion modes for photothermally responsive hydrogels. A strip that undergoes reversible bending normally returns along approximately the same deformation path and would produce little net translation on an ideal, frictionally uniform surface. Directional crawling therefore requires a difference between the forward and recovery strokes. This difference may originate from geometric asymmetry, directional surface textures, state-dependent adhesion, spatially scanned illumination, or hysteresis between hydrogel deformation and interfacial friction.

Figure 8A presents the original locomotion experiment reported by Zhao et al., rather than a compilation of previously published actuator images. A PNIPAm–4-hydroxybutyl acrylate hydrogel strip was prepared hydrothermally, and graphene oxide was introduced into a selected region to provide photothermal activity. Near-infrared irradiation caused the central region of the strip to bend, whereas removal of the light resulted in partial shape recovery. Because the forward bending stroke and reverse recovery stroke were mechanically unequal, their displacements did not cancel. The strip consequently accumulated a net forward displacement of approximately 20 mm after two irradiation cycles in the reported experiment [82]. This example shows that a simple bending actuator can crawl without articulated legs when the position of the active region and the deformation history provide sufficient asymmetry.

A different strategy is illustrated in Figure 8B. Liu and Zheng fabricated a caterpillar-inspired anisotropic bilayer based on PNIPAm–poly(vinyl alcohol) and PNIPAm–sodium alginate networks, with Fe_3_O_4_ particles incorporated to improve photothermal conversion and thermal transport. On a ratchet-patterned substrate, illumination progressively bent the robot until its leading edge engaged with a ratchet tooth; continued bending then pulled the body forward. The substrate therefore acted as an external mechanical rectifier [83]. The response should nevertheless be described on the timescale reported in the source. For the optimized Fe_3_O_4_-containing formulation, the time required to reach a bending angle of approximately 30° was about 30 min, compared with roughly 1 h for the reference material. After the light was removed, cooling, water uptake, and recovery to the initial configuration required about 6 min. Thus, the term “fast response” in the original article denotes an improvement relative to the authors’ control formulations, rather than a generally rapid response compared with microscale photothermal hydrogel robots. The reported ground speed of 54.5 mm h^−1^ also reflects the long actuation–recovery cycle and the large displacement produced by each ratchet-assisted step.

Figure 8C demonstrates peristaltic locomotion at a substantially smaller scale. Zhang et al. fabricated a PNIPAM–single-walled carbon nanotube microrobot from truncated-octahedron lattice cells. A moving laser spot induced sequential local shrinkage and reswelling along the body. The swollen and contracted regions exhibited different contact conditions, so that the optical scan redistributed friction between the front and rear sections and generated a six-stage peristaltic cycle [80]. The porous lattice shortened solvent-transport distances and lowered the relative density, producing a maximum reported peristaltic speed of 15.15 μm s^−1^, equivalent to 0.14 body lengths per second. Compared with a solid hydrogel microrobot of identical external dimensions, the lattice design required markedly less optical energy and responded more rapidly.

The lamellar hydrogel in Figure 8D further illustrates how internal anisotropy can couple photothermal strain to frictional switching. Yao et al. confined a PNIPAM/CNT cellular network within an aligned Ag-nanowire/sodium-alginate lamellar scaffold. During sequential NIR scanning, the illuminated region contracted and changed from a relatively hydrophilic to a more hydrophobic interfacial state. Subsequent cooling and rehydration reversed both the dimensional strain and the interfacial condition. Alternating contraction, expansion, and frictional anchoring caused the hydrogel to crawl opposite to the scanning direction [84]. Because the contraction force was primarily oriented perpendicular to the lamellae, the angle between the lamellae and the robot axis determined whether the body translated, turned, or rotated. This system is therefore notable for encoding locomotion direction in the material architecture rather than relying exclusively on the incident-light direction.

Several additional studies clarify the role of friction in light-driven crawling. Rehor et al. developed approximately 100 μm PNIPAM microcrawlers containing gold nanoparticles. Off-centre laser pulses caused local collapse and reswelling, while hysteresis between deformation and substrate friction produced directional displacement and enabled the robots to push small cargo [85]. Kropacek et al. subsequently separated propulsion and steering into different robot regions: a gold-loaded PNIPAM tail generated cyclic movement under spatially homogeneous illumination, whereas a UV-responsive front section controlled turning [11]. At a larger scale, Li et al. reinforced a spiropyran-functionalized covalent hydrogel with aligned peptide-amphiphile supramolecular fibres. The aligned internal skeleton accelerated water transport, enhanced bending, and increased the crawling step length of a four-footed actuator under visible light [47]. More recently, multimaterial printing of tough, light-responsive hydrogel fibres has enabled walking, turning, flipping, and cargo transport through the combined design of printed geometry and spatiotemporal illumination [86].

Taken together, the systems in Figure 8 show three progressively more integrated approaches. Ratchets and asymmetric outlines rectify otherwise reciprocal bending at the device or substrate level; scanned illumination imposes a travelling deformation wave; and anisotropic or porous networks couple actuation directly to a state-dependent friction field. The latter two approaches offer finer steering and miniaturization, but their performance remains sensitive to substrate roughness, hydration, optical alignment, and heat dissipation. Reporting only displacement or nominal velocity is consequently insufficient. Actuation time, recovery time, light intensity, robot length, substrate condition, and displacement per cycle should be provided together for meaningful comparison.

### 5.2. High-Amplitude Locomotion of Polymeric Soft Robots

Jumping and rolling require stronger symmetry breaking than quasi-static crawling. Jumping generally depends on rapid storage and release of elastic or interfacial energy, whereas continuous rolling requires repeated relocation of the illuminated, load-bearing, or high-friction region. These modes can produce faster displacement and improved obstacle-crossing ability, although they often require higher irradiance or more elaborate body geometry.

Figure 9A shows the light-driven hydrogel developed by Li et al. for rapid in-air jumping and rolling. The actuator consisted of a poly(sodium acrylate) hydrogel containing iron oxide nanoparticles. Focused irradiation generated local photothermal heating and vaporization of water retained inside the hydrogel. Bubble nucleation and growth produced a rapid deformation impulse, while elastic recoil converted the transient pressure into take-off or rolling [18]. The reported response time was approximately 800 ms, with take-off velocities up to 1.6 m s^−1^ and jumping heights of approximately 15 cm. By changing its configuration and irradiation position, the same actuator jumped over barriers, rolled on inclined surfaces, passed through confined openings, and combined rolling and jumping to negotiate a bent channel. In this case, the hydrogel functions both as a water reservoir and as an elastic body, while the immediate propulsive event is produced by a localized liquid–vapour transition.

Continuous motion under constant light can also arise from geometric feedback rather than externally programmed light pulses. Figure 9B summarizes the hydrogel knotbots developed by Zhu et al. An anisotropic photothermal hydrogel ribbon was tied into torus knots with different topologies. Light-induced strain, stored prestress, self-contact, and periodic self-shadowing repeatedly changed which segment was exposed to light [78]. The topology therefore provided an internal feedback loop: deformation moved the active region out of illumination and exposed another segment, sustaining rolling or spinning without time-varying external control. The rolling direction and trajectory depended on both knot topology and the direction of illumination.

Figure 9C represents a molecularly programmed route to motion reversal. Guo et al. incorporated two spiropyran derivatives with different photoisomerization kinetics and charge responses into a chemically homogeneous hydrogel. Under constant illumination, the combined network charge first decreased and then increased, giving a sequential contraction–expansion response [87]. Ring-shaped hydrogel robots consequently rolled in one direction before reversing without switching the incident light. The result is important because temporal complexity was encoded at the molecular level rather than through layered fabrication, moving light patterns, or closed-loop electronic control.

In Figure 9D, the lattice microrobot introduced in Figure 8C is operated at higher laser power. At 200 mW, irradiation near the tail generated a strong local temperature gradient and thermophoretic propulsion, producing continuous hopping rather than surface-bound peristalsis [80]. The average and maximum hopping speeds were 9.36 and 20.63 μm s^−1^, respectively. Off-axis irradiation introduced a torque, enabling a reported turning angle of 73.89° and subsequent navigation through a patterned maze. This faster gait involved a clear energetic penalty: its reported energy-conversion efficiency was only 39.46% of the peristaltic mode, despite a speed approximately 1.26 times higher and an optical-energy input four times greater.

Recent hydrogel designs further demonstrate that continuous rolling need not rely on a knot. An anisotropic cylindrical hydrogel can break mirror symmetry through a photothermal strain gradient and continuously expose a fresh region as it rolls. Such robots reached 1.27 mm s^−1^ on a horizontal submerged surface and climbed an 18° ramp at 0.57 mm s^−1^ under constant illumination [88]. Closed twisted ribbons, including Möbius and Seifert configurations, similarly use topology, rapid reversible deformation, and self-shadowing to support continuous flipping and rotation [89]. By contrast, self-rolling nanocomposite hydrogel tubes containing magnetic nanorods represent a hybrid case: NIR light opens the tube or changes its diameter, whereas an applied magnetic field supplies translational guidance [90].

These studies identify three routes to non-reciprocal high-amplitude locomotion. The first converts optical energy into a short impulse through bubble formation or rapid thermophoretic flow. The second encodes feedback in topology, curvature, and self-shadowing. The third programs a non-monotonic material response through molecular kinetics. Impulsive methods provide excellent obstacle-crossing capability but introduce thermal and trajectory-control challenges. Topological rolling requires less temporal control but is sensitive to contact, self-shadowing, and initial configuration. Molecularly programmed reversal reduces dependence on external modulation, although chemical recovery and photoswitch fatigue may limit operating frequency.

### 5.3. Bio-Inspired Underwater Locomotion of Responsive Hydrogels

At low Reynolds numbers, a swimmer executing a single reciprocal shape change cannot generate sustained net translation in an unbounded Newtonian fluid. Light-responsive hydrogel swimmers therefore use travelling waves, non-equilibrium swelling cycles, asymmetric body geometries, oscillatory appendages, interfacial gradients, or flow fields. Figure 10 covers this range from centimetre-scale fish-like robots to microscale helical swimmers.

Figure 10A shows a multifunctional fish-like soft robot constructed by Wang et al. DMD-based photopolymerization was used to fabricate PNIPAM structures, after which carbon nanotubes were introduced to provide photothermal responsiveness. The tail contained a flexible hydrogel drive unit integrated with a fin, while the remaining fish body and balance structures were manufactured separately [63]. Alternating illumination drove tail oscillation and forward swimming. A separately addressed NIR-responsive arm and micromanipulator enabled grasping, object transport, and release. This is therefore an integrated soft-robotic platform in which hydrogel actuators power selected appendages rather than constituting the entire robot body.

Figure 10B presents the pulsed microswimmer reported by Mourran et al. The swimmer was a tapered PNIPAM ribbon, 80 or 120 μm long, coated on one side with an approximately 2 nm gold layer and containing dispersed gold nanorods. In cold water, the wedge-shaped bilayer adopted a conical helical state; heating above the PNIPAM transition extended it toward a filament-like configuration [91]. Millisecond NIR pulses created a non-equilibrium coil–uncoil cycle in which heating, cooling, and solvent migration occurred on different timescales. The resulting sequence was non-time-reversible and generated propulsion. Under optimized pulse conditions, the microswimmer reached approximately 500 μm s^−1^, or six body lengths per second. Pulse duration and recovery time also shifted the motion between forward swimming and localized spinning.

Figure 10C shows a related microgel rotor. A PNIPAM-based bilayer ribbon with plasmonic heating components was formed into a spiral or helical structure, while a microsphere at one end served as a pivot or hydrodynamic anchor. Pulsed irradiation generated cyclic unwinding and recoiling, producing rotation around the anchored end [92]. This device should therefore be described as a light-driven rotor rather than an untethered translating swimmer. Its significance lies in converting out-of-equilibrium hydrogel deformation into controlled rotational motion and local fluid circulation.

The system in Figure 10D occupies another intermediate category. Cao et al. prepared green-light-responsive P(NIPAM-co-acrylamide)/Fe_3_O_4_ hydrogel actuators and demonstrated that a boomerang-shaped actuator could propel a floating foam platform [93]. The hydrogel changed shape under 520 nm irradiation and mechanically drove the floating structure. The demonstrated object was therefore not a freely swimming all-hydrogel robot, but a hydrogel-powered surface vehicle. This distinction is relevant when comparing propulsion efficiency and payload capacity, because buoyancy and the hydrogel–foam interface contribute directly to the observed motion.

Figure 10E illustrates a reprogrammable underwater robot developed by Ni et al. Dynamic disulfide exchange was used to photo-mechanically program spatially selective anisotropy into a thermoresponsive PNIPAM network. Incorporation of photothermal fillers then enabled rapid light-powered actuation [16]. Unlike conventional diffusion-dominated PNIPAM gels, deformation was governed mainly by thermally induced conformational changes of locally oriented chains. Oscillation frequencies up to 1.7 Hz were achieved, and the same hydrogel specimen could be reprogrammed to swim, crawl, or rotate. The study therefore connects reversible network programming with gait selection, rather than assigning a permanent structure to each locomotion mode.

Earlier nonequilibrium microgels containing plasmonic gold nanorods established that a delay between rapid photothermal heating and slower volume relaxation can generate complex cyclic deformation and propulsion [94]. At a larger scale, the OsciBot converted constant visible illumination into self-sustained oscillation through a self-shadowing negative-feedback loop. The resulting hydrogel oscillator produced directional, phototactic swimming without externally imposed light pulses [8]. Hou et al. subsequently demonstrated a hydrogel-based underwater phototactic vehicle in which chemomechanical deformation, photothermal heating, and the surrounding flow formed a closed feedback loop. The vehicle adjusted its pose, generated directional flow, and followed the incident-light direction in three dimensions [28].

The systems in Figure 10 show that “swimming” includes several mechanically distinct situations: direct body-wave propulsion, tethered rotation, hydrogel-actuated propulsion of a passive platform, and hydrogel–fluid feedback. A useful comparison must therefore specify whether the hydrogel itself translates freely, whether an external support or buoyant platform is present, whether the illumination is pulsed or constant, and whether propulsion originates predominantly from body deformation or from a light-induced flow field.

### 5.4. Multimodal Locomotion in Hybrid and Multi-Field Responsive Gel Systems

Multimodal operation requires more than changing the direction of a single gait. A robot must switch between mechanically distinct behaviours, reconfigure its body, selectively actuate multiple joints, or combine locomotion with manipulation, assembly, sensing, or cargo delivery. Light is particularly valuable in this context because spatially patterned illumination can address different regions without adding wired connections. Nevertheless, several prominent examples combine light with magnetic or electric fields; these should be identified as hybrid systems rather than classified as purely light-propelled hydrogel robots.

Figure 11A shows the multi-joint microactuator fabricated by Xin et al. Two-photon polymerization generated asymmetric hydrogel joints, and femtosecond-laser photoreduction deposited Ag nanoparticles at selected locations. The Ag nanoparticles converted focused light into local heat, allowing individual joints to be addressed independently with patterned multifocal illumination [69]. Response times of approximately 30 ms and actuation powers below 10 mW were reported. By coordinating two joints, a microscale robotic arm collected particles from different positions. The principal advance was not long-range locomotion but the conversion of spatially resolved light into selective, multi-degree-of-freedom manipulation.

Figure 11B extends this strategy to reconfigurable collectives. Ren et al. fabricated ant-inspired microbots consisting of magnetic photoresist bodies, thermoresponsive asymmetric hydrogel joints, and photoreduced Ag nanoparticles near the mandibles [70]. NIR irradiation opened the mandibles by heating the hydrogel joints, enabling reversible assembly and release, whereas magnetic fields supplied body translation and orientation. Three connected units crossed a gap that single or double units could not traverse. In a maze, the collective changed configuration, transported a doxorubicin-loaded hydrogel cube, and released it near HeLa cells. This is a light–magnetic hybrid system: light controls gripping and structural reconfiguration, while magnetic forces provide the principal locomotion. Figure 11C returns to a monomaterial, topology-mediated platform. Hydrogel knotbots were used not only for rolling but also for climbing and mechanical work. By interacting with cylindrical supports and rings, the knotbots rotated a gear, climbed a threaded rod, translated along a smooth rod, and transported or rotated a passive object [78]. These demonstrations show how topological self-contact can function as a transmission element, transforming distributed photothermal deformation into object-scale rotation, climbing, or cargo movement.

Figure 11D should be classified explicitly as a hybrid photochemical–electrophoretic system. Yang et al. incorporated two spiropyran derivatives into a PNIPAM hydrogel so that blue light altered the network’s net charge and eventually reversed its sign [95]. The translational force, however, was generated primarily by electrophoresis in an applied direct-current electric field. Light regulated the sign and timing of the electrokinetic response, while dielectric perturbations enabled cargo capture, obstacle avoidance, delivery, and return motion. The hydrogel therefore acts as a light-programmable electroactive robot, not as a body propelled directly by light-induced swelling or bending. Figure 11E represents an even broader boundary case. Hou et al. prepared a tough rGO/polyacrylamide hydrogel robot whose network exhibited negligible photo-induced mechanical deformation [96]. Absorption of light by rGO produced a localized thermal field, which drove fluid motion around the body and enabled translation, swimming, obstacle negotiation, and escape from confined regions. The reported speed reached 1.27 cm s^−1^. Because the hydrogel body is mechanically photo-inactive, this platform should be classified as a photo-thermo-fluidically propelled gel robot rather than a conventional light-responsive hydrogel actuator. Its relevance lies in demonstrating that hydrogel robots can exploit surrounding-fluid dynamics without repeatedly swelling or contracting their networks.

Other hybrid and modular systems reinforce this expanded interpretation of task execution. Hydrogel–metal composites containing aligned ferromagnetic nanowires can be reshaped by light and translated or oriented magnetically, enabling walking, climbing, cargo transport, and programmed release [9]. Vrba et al. developed an automated platform that located and drove individual AuNP-containing PNIPAM microgels with focused laser pulses, assembled them into a prescribed arrangement, and chemically bonded the units with local UV exposure. The resulting structures could push and deposit cargo [97]. Kropacek et al. further showed that mobile hydrogel microcrawlers could self-assemble under confinement, be bound into porous sheets by light, and subsequently undergo programmed three-dimensional bending, locomotion, and manipulation [98]. The 3D-printed tough hydrogel robots discussed above likewise combined walking, turning, flipping, and cargo transport within one printed material platform [86].

The systems shown in Figure 11 indicate that multimodal operation should be classified according to the actual role of light in energy input and control. In directly actuated systems, photo-induced swelling, contraction, or bending of the hydrogel produces both deformation and propulsion. These systems have comparatively simple compositions and are useful for establishing direct relationships among material response, structural deformation, and locomotion, but their speed, force output, and operating distance remain constrained by heat transfer, mass transport, and optical attenuation. In light–magnetic or light–electric hybrid systems, optical stimulation primarily controls gripping, adhesion, charge state, or structural reconfiguration, whereas magnetic or electric fields provide long-range translation and orientation. This functional division combines the local addressability of light with the navigational capabilities of magnetic or electric actuation, but it requires additional equipment and control variables, and the resulting locomotion cannot be attributed entirely to photo-induced hydrogel deformation. Photothermofluidic systems further broaden the definition of a hydrogel robot: the hydrogel primarily absorbs light and regulates the thermal field, whereas propulsion originates from motion of the surrounding fluid. This approach may reduce fatigue associated with repeated volumetric deformation, but the trajectory becomes sensitive to container boundaries, fluid properties, and temperature distribution.

The principal advantage of light-responsive hydrogels over other soft-robot actuation methods is therefore not that they invariably provide the highest speed or force, but that they offer distinctive capabilities when non-contact local addressing, independent control of multiple regions, wavelength selection, or coupling between deformation and functional release is required. In deep tissue, optically turbid media, or large-scale load-bearing conditions, purely optical actuation may not be the most practical option, and hybrid-field operation can provide a more realistic solution. Future studies should report optical intensity, wavelength, irradiation area, response and recovery times, displacement or velocity, body-length-normalized speed, output force, cycle life, environmental conditions, and the contribution of auxiliary fields. Direct and hybrid systems can only be compared fairly when the contributions of the different energy inputs are clearly separated. We anticipate that genuinely task-autonomous systems will move beyond manually switching multiple light spots and will instead combine material-level feedback, real-time localization, and closed-loop optical control to adjust locomotion in response to environmental changes.

## 6. Sensing, Manipulation, and Biomedical Applications of Light-Responsive Hydrogel-Driven Soft Robots

**Figure 12:**
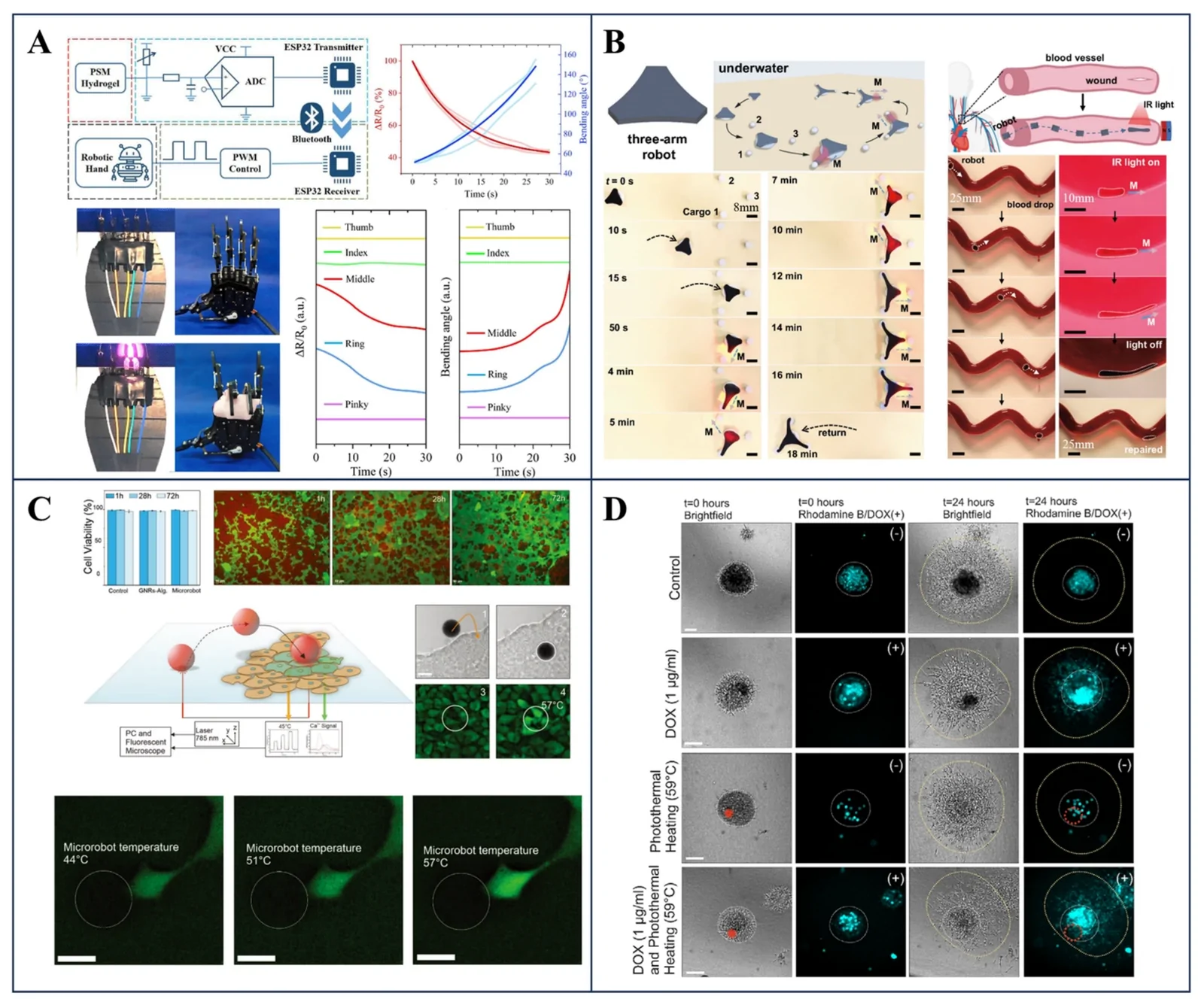
Sensing, manipulation, and biomedical applications of light-responsive hydrogel-driven soft robots. (**A**) A photothermally actuated gradient hydrogel simultaneously produces deformation-dependent resistance signals, enabling remote interaction between hydrogel actuators and a robotic hand [58]. (**B**) A Fe_3_O_4_-containing protein hydrogel robot combines infrared-regulated adhesion and morphing with magnetic navigation for underwater cargo manipulation and proof-of-concept repair of an artificial blood vessel [101]. (**C**) An alginate-based microrobot containing gold nanorods and Rhodamine B enables laser-driven locomotion, localized thermal stimulation of single cells, and fluorescence-based temperature measurement [104]. (**D**) A next-generation alginate microrobot supports cell transport and spheroid assembly and enables combined photothermal and chemotherapeutic screening in three-dimensional tumour models [105]. Representative schematics and experimental images were adapted from the cited open-access sources.

With the principal locomotion strategies established, research on light-responsive hydrogel robots is shifting from demonstrations of deformation and mobility toward perception, manipulation, and task-specific operation. Here, self-sensing denotes the conversion of deformation or environmental interaction into a measurable signal that can support state estimation or feedback control. Biomedical relevance likewise requires more than material biocompatibility; the robot must perform a defined operation, such as manipulating cells, delivering cargo, regulating a local microenvironment, or interacting with a tissue model. Figure 12 summarizes this progression from integrated actuation and sensing to underwater manipulation, single-cell stimulation, and three-dimensional drug screening.

The development of light-responsive hydrogel robots is gradually shifting from the demonstration of deformation and locomotion toward the integration of perception, manipulation, and task-specific functions. In this context, sensing should not be regarded merely as the optical observation of robot motion. A genuinely self-sensing hydrogel robot must convert its deformation or environmental interaction into a measurable signal that can subsequently be used for state estimation or feedback control. Likewise, biomedical relevance requires more than material biocompatibility: the robot must accomplish a defined operation, such as manipulating cells, delivering cargo, regulating a local microenvironment, or interacting with tissue models. Figure 12 summarizes this progression from self-sensing actuation and robotic interaction to underwater manipulation, single-cell stimulation, and three-dimensional drug screening.

Figure 12A presents a representative strategy for integrating actuation and strain sensing within the same hydrogel body. Liu et al. developed a multifunctional gradient hydrogel containing photothermally active MoO_2_ nanosheets. The asymmetric network structure was produced through the rapid precipitation of the nanosheets, generating a through-thickness difference in hydrophilicity and therefore an internal actuation gradient. Under 808 nm irradiation, photothermal heating triggered asymmetric deswelling and bending. At the same time, deformation altered the electrical resistance of the conductive hydrogel, enabling the bending state to be inferred from the relative resistance change. Five hydrogel actuators were subsequently coupled to an Internet-of-Things control system, through which the measured resistance signals were mapped to the bending angles of the fingers of a robotic hand [58]. This arrangement is important because the sensing element is not simply attached to the actuator; actuation and sensing originate from the same continuous material, reducing interfacial mismatch and improving the correspondence between local strain and the electrical output.

Similar integration has been achieved using conductive-polymer networks embedded in thermoresponsive hydrogels. Lo et al. combined PNIPAAm with a light-absorbing and electrically conductive polypyrrole network, allowing near-infrared irradiation to induce hydrogel contraction while changes in the conductive pathways reported the resulting strain [99]. An ice-templated conductive hydrogel was subsequently used to combine photothermal actuation, strain and pressure perception, object recognition, grasping, and feedback-regulated stepwise motion in a monolithic somatosensory actuator [100]. These studies demonstrate that electrical conductivity in a photoresponsive hydrogel can support three functions simultaneously: photothermal energy conversion, deformation sensing, and signal transmission.

More recent systems have extended sensing beyond proprioception. The moiré-superlattice hydrogel robot reported by Zhang et al. combined a PNIPAM matrix with a BP/WS_2_ functional layer. Photothermal contraction drove locomotion, whereas photoelectric signals generated by the same functional material were used to detect an external infrared heat source [14]. This approach separates the actuation and sensing wavelength ranges and therefore reduces interference between robot propulsion and environmental detection. At the device level, Zheng and Jia developed a photoresponsive hydrogel soft lens in which an rGO-containing PNIPAM actuator changed the geometry of a soft optical element in response to incident light. The resulting focal adjustment, wavefront modulation, and optical steering illustrate how responsive hydrogels can form part of a soft robotic perception system rather than merely serving as mechanical actuators [12].

It is nevertheless necessary to distinguish material-level self-sensing from externally observed feedback. Camera-based tracking, infrared thermography, and microscopy can close a control loop without giving the hydrogel intrinsic sensory capability. For example, the homogeneous-illumination microcrawler discussed in Section 5.1 relied on external observation to steer its reversibly deformable front section, whereas the automated assembly platform discussed in Section 5.4 combined image-based localization, laser positioning, and trajectory planning to organize multiple light-powered hydrogel microrobots into predefined formations. These systems demonstrate closed-loop optical control, but their feedback signals are supplied by external imaging apparatus. Accordingly, self-sensing materials, externally sensed robots, and fully closed-loop robotic systems should be treated as related but distinct levels of functional integration.

In addition to sensing, light-responsive hydrogels can be combined with complementary driving fields to perform manipulation tasks that cannot be accomplished by photothermal deformation alone. Figure 12B shows the protein-hydrogel robot developed by Huang et al. A positively charged resilin-like protein was complexed with polyanionic polyoxometalates to produce a hydrogel capable of switching between a soft, adhesive state and a comparatively stiff, non-adhesive state over a narrow temperature interval. The incorporation of Fe_3_O_4_ nanoparticles introduced photothermal responsiveness and magnetic controllability. Infrared irradiation regulated hydrogel softening, adhesion, and shape morphing, whereas an external magnetic field provided navigation and directional control. The resulting three-arm robot captured and transported multiple objects underwater and was guided through an artificial blood-vessel model to cover a leakage site [101]. Because locomotion was principally magnetically directed while light regulated the mechanical and adhesive state, this system should be classified as a light-responsive, magnetically navigated hybrid hydrogel robot rather than a purely light-propelled robot. Moreover, the vessel-repair experiment was conducted in an artificial model and should be presented as a proof of concept rather than an in vivo therapeutic demonstration.

Koike et al. used light-absorbing micropatterns beneath PNIPAAm microactuators to localize heating on a microfluidic chip. Ring- and frame-shaped actuators could be individually addressed for cell trapping, mechanical stimulation, and release without electrical wiring at every actuation site [102]. The subsequent incorporation of a digital micromirror device enabled real-time patterned irradiation and improved the selectivity with which densely integrated hydrogel microactuators could be addressed [103]. These studies are especially relevant to high-throughput cellular manipulation because the light pattern acts as a reconfigurable control layer, whereas the physical actuator array can remain unchanged.

Figure 12C shows the thermally activated cell-signal imaging microrobot developed by Harder et al. The robot consisted of a calcium-crosslinked alginate microgel containing gold nanorods and covalently incorporated Rhodamine B. Gold nanorods converted 785 nm irradiation into localized heat, whereas the temperature-dependent fluorescence intensity of Rhodamine B provided real-time thermometry. Laser-induced thermal gradients also generated fluid motion that enabled the microrobot to move in three-dimensional workspaces. The platform was positioned near selected mammalian cells and used to produce controlled local heating, allowing temperature-dependent calcium signalling to be investigated while cell viability was monitored [104].

This example requires a precise mechanistic classification. The alginate body in Figure 12C functions as a biocompatible carrier for the photothermal transducer and fluorescent thermometer, but its locomotion does not arise primarily from light-induced swelling or bending of the hydrogel network. Instead, motion is generated by optically induced thermophoretic convection in the surrounding fluid. It is therefore a hybrid photo-thermo-fluidic hydrogel microrobot and constitutes a boundary case within a review of deformation-driven hydrogel robots. Its relevance lies in demonstrating how a hydrogel robot can unite mobility, localized stimulation, and in situ measurement even when the hydrogel is not itself the principal mechanical motor.

Figure 12D represents the subsequent extension of this platform to three-dimensional cell models. Harder et al. modified alginate microgels to support cell attachment, single-cell transport, and controlled spheroid assembly. The incorporation of gold nanorods preserved localized photothermal stimulation, whereas Rhodamine B supplied fluorescence-based temperature feedback. This system was used to examine the response of fibrosarcoma spheroids to localized heating, doxorubicin, and their combination [105]. Under the reported experimental conditions, combined photothermal and chemotherapeutic treatment reduced cell viability and invasive behaviour more effectively than either treatment alone. The work therefore advances the role of hydrogel microrobots from cargo manipulation toward active regulation and measurement of three-dimensional cellular microenvironments. At the present stage, however, it remains an in vitro drug-screening platform rather than an in vivo therapeutic robot.

Light-triggered drug release provides another route toward biomedical implementation. Lee et al. developed a spring-shaped alginate/NIPAM hydrogel microrobot containing magnetic nanoparticles and doxorubicin. Electromagnetic fields were used for targeting, while near-infrared heating caused swelling–deswelling changes that regulated drug release [106]. Kim et al. subsequently reported a gelatin/PVA hydrogel microrobot containing magnetic nanoparticles and PLGA–doxorubicin particles. Following magnetic positioning, near-infrared irradiation decomposed the hydrogel structure; the magnetic particles could then be retrieved while the drug-loaded particles remained at the target location [107]. These systems highlight the value of separating navigation and therapeutic activation: magnetic fields provide comparatively deep and directional control, whereas light offers localized release with high temporal resolution.

It should be emphasized that the biomedical studies discussed in this section and the representative systems shown in Figure 12 have not yet been employed in vivo animal models. Current validation has mainly relied on artificial vascular channels, cultured mammalian cells, and three-dimensional tumour spheroids. These platforms are suitable for evaluating localized temperature regulation, cellular stimulation, cargo release, and short-term cytocompatibility, but they do not fully reproduce blood-flow shear, tissue scattering, immune responses, protein adsorption, material metabolism, or long-term clearance in a living organism. The corresponding results are therefore described here as proofs of concept or in vitro functional demonstrations rather than as therapeutic platforms validated in animals.

Future animal-model selection should be matched to the intended application, robot dimensions, and target site and should follow a staged validation pathway from flow phantoms, ex vivo tissues, and three-dimensional cellular systems to in vivo studies. Before animal experiments are initiated, navigation accuracy, maximum local temperature, optical dose, degradation products, residence time, and retrieval strategy under physiological conditions should be established. Only after these parameters are reliably controlled can an animal model provide a meaningful assessment of therapeutic potential rather than merely reproduce locomotion observed in a static liquid.

Despite rapid functional progress, most light-responsive hydrogel robots remain at the level of controlled laboratory demonstrations. Optical attenuation and scattering limit the delivery of sufficient energy to deep tissue, even when near-infrared wavelengths are used. Increasing irradiation power can accelerate actuation but may also enlarge the heated region and raise the risk of nonspecific thermal damage. For biomedical studies, laser wavelength, power density, exposure duration, maximum local temperature, irradiation area, and distance between the photothermal element and cells should therefore be reported together rather than treating irradiation power as an isolated parameter.

Material safety must also be examined over time scales longer than a single actuation experiment. The cytocompatibility of the pristine hydrogel does not by itself establish the safety of the complete robot. Photothermal nanoparticles, conductive polymers, fluorescent dyes, unreacted monomers, and degradation products may diffuse from the network or alter cellular behaviour. Physiological ionic strength, proteins, enzymes, and repeated swelling cycles may further change adhesion, phase-transition temperature, electrical calibration, and mechanical integrity. Biodegradable designs and strategies for retrieving persistent magnetic or plasmonic components are therefore particularly important for future in vivo applications.

Finally, biomedical autonomy will require the coordinated integration of navigation, state estimation, and dose control. Electrical resistance and fluorescence thermometry offer promising routes for proprioception and local temperature feedback, respectively, but both are susceptible to drift, hysteresis, and environmental interference. Reliable closed-loop operation will require calibration models that distinguish deformation, temperature, hydration, and ionic effects. Progress toward clinically relevant systems should consequently be assessed through task-level metrics—including targeting accuracy, manipulation success rate, thermal dose, cargo-release kinetics, cell viability, recovery or degradation, and performance in flowing biological media—rather than deformation amplitude alone.

## 7. Current Challenges

Despite substantial advances in responsive materials, structural programming, and locomotion control, light-responsive hydrogel soft robots still face several interconnected challenges that limit their transition from laboratory demonstrations to reliable functional systems. Optical attenuation and scattering reduce the energy delivered to active regions, particularly in thick hydrogels, particle-rich composites, turbid liquids, and biological tissues. Increasing photothermal-agent concentration or irradiation intensity can accelerate heating, but may also decrease optical penetration, create non-uniform temperature fields, promote dehydration, and increase the risk of thermal damage.

Optical attenuation should be considered as a coupled transport constraint rather than solely as a loss of incident power. In hydrated polymer networks, the delivered optical dose is determined by wavelength-dependent absorption, scattering, scattering anisotropy, and refractive-index heterogeneity. These parameters vary with water content, chromophore concentration, particle dispersion, and the optical properties of the surrounding medium; in biological tissues, endogenous absorbers and heterogeneous microstructures introduce an additional dependence on wavelength and propagation depth. Moreover, deswelling can increase polymer density and refractive-index contrast, so that a hydrogel that is initially accessible to light may become more strongly scattering during actuation. The resulting self-screening produces surface-biased heating, delayed activation of internal layers, and a reduction in the effectively actuated volume. For robotic systems, these effects may decrease deformation amplitude and force output, broaden the addressed region, introduce cross-talk between adjacent joints, and increase trajectory variability. Simply increasing the source irradiance is therefore not a general solution, because it can overheat the illuminated surface before the required optical dose reaches deeper regions. Mitigation should instead combine wavelength selection based on the measured absorption and scattering spectra of the complete operating system, reduction of the optical path length through thin, porous, lattice, or microscale structures, and controlled spatial distribution of the photothermal component. Bidirectional or multi-angle illumination may reduce depth-dependent thermal gradients, whereas scanned or pulsed exposure can limit local heat accumulation. Closed-loop control based on local temperature, deformation, or transmitted-light measurements is particularly important when the optical properties evolve during the phase transition. For meaningful comparison, incident irradiance should be accompanied, where possible, by spectral transmittance, attenuation length or transmitted fluence, beam dimensions at the actuator, internal temperature distribution, and the optical state of the hydrogel before and after actuation.

Plasmonic materials provide a particularly instructive example of both the opportunities and limitations of optical-energy delivery. Gold nanorods and related nanostructures can convert light into heat through localized surface plasmon resonance, allowing the absorption band to be adjusted through particle geometry and enabling wavelength-selective activation. However, the plasmonic response is sensitive to nanoparticle aggregation, surface chemistry, local refractive index, and interparticle spacing. Consequently, hydrogel swelling or collapse may shift or broaden the resonance while simultaneously altering bulk scattering. In a thermoplasmonic hydrogel attenuator, gold nanorods resonant near 810 nm activated a copolymer transition above approximately 37 °C, producing an optical contrast of about 65% and a plasmon-band shift of approximately 30 nm; these observations demonstrate that the optical transfer function can change substantially during the response itself [108]. A complementary strategy integrated gold-nanorod heaters with fluorescent temperature probes in a three-dimensional hydrogel matrix, permitting localized plasmonic actuation and spatial monitoring of the accompanying temperature field [109]. More recently, control of gold-nanorod characteristics and excitation wavelength has been used to obtain selectively addressable photothermal deformation based on coupled plasmonic effects [110]. These studies suggest that plasmonic transducers are most effective when their resonance is matched to the complete hydrogel–medium optical window and when particle loading, dispersion, and placement are optimized jointly. Immobilizing the nanoparticles within the network, restricting them to active joints, and combining spectrally selective excitation with temperature feedback can reduce aggregation, unnecessary bulk absorption, and thermal cross-talk. Nevertheless, plasmonic enhancement does not remove attenuation in thick or turbid environments; it redistributes where optical energy is absorbed. Its benefit should therefore be evaluated through the depth and spatial precision of functional actuation, rather than photothermal conversion efficiency alone.

Moreover, rapid molecular transitions do not necessarily produce rapid robotic motion because macroscopic deformation often depends on slower heat transfer and water migration. This creates an inherent compromise among actuator size, response speed, deformation amplitude, force output, and recovery time. Structural complexity introduces additional difficulties. Bilayers may undergo interfacial fatigue or delamination, whereas continuous gradients depend strongly on the reproducibility of particle sedimentation, diffusion, and polymerization. Three-dimensional printing and femtosecond-laser processing provide precise spatial control but remain constrained by fabrication throughput, exposure-dependent crosslinking, dimensional changes after swelling, and batch-to-batch variation. Long-term operation may also be affected by network fatigue, curvature drift, particle aggregation, and the leaching of functional components. Comparison among reported robots remains difficult because actuation performance is measured under different wavelengths, optical intensities, robot dimensions, environmental conditions, and definitions of response time. Standardized reporting should therefore include the irradiation conditions, actuator dimensions, response and recovery times, body-length-normalized velocity, output force or payload, cycle life, operating medium, and maximum local temperature. Mechanistic distinctions are equally important: robots driven directly by photo-induced hydrogel deformation should be differentiated from hybrid systems in which light regulates adhesion, charge, stiffness, or configuration while magnetic, electric, or fluidic effects provide propulsion. In addition, most current platforms rely on manually controlled illumination or external imaging, while intrinsic sensing signals remain susceptible to hysteresis, drift, hydration, and ionic interference. Biomedical translation presents further challenges because the systems reviewed here have mainly been evaluated in artificial channels, cell cultures, and three-dimensional spheroids rather than in vivo animal models. Tissue scattering, flow-induced forces, immune responses, degradation, component retrieval, and the biological consequences of repeated photothermal exposure remain insufficiently characterized. These limitations are closely coupled; improving one performance parameter may compromise another. Accordingly, progress should be evaluated through repeatable task completion under clearly defined optical, mechanical, and environmental conditions rather than through maximum deformation or locomotion speed alone.

To make these trade-offs explicit, Table 1 compares representative light-responsive hydrogel robotic systems using a common set of performance descriptors. The fastest absolute motion was obtained by bubble-mediated jumping in air, whereas the highest body-length-normalized translation was reported for a pulsed helical microswimmer. By contrast, diffusion-limited terrestrial crawlers operated more slowly but achieved large step lengths with simple centimetre-scale bodies, while lattice architectures improved deformation rate and reduced optical energy relative to solid controls. Low-power femtosecond-written microjoints provided rapid local deformation but required high-precision optical fabrication and addressing. Systems integrating sensing, adhesion, or cell stimulation introduced task-specific figures of merit—such as gauge factor, adhesion strength, temperature resolution, and manipulation capability—that cannot be reduced to locomotion speed alone. Accordingly, the table is intended as a condition-aware comparison rather than a rank order: robot dimensions, medium, illumination geometry, boundary conditions, and contributions from magnetic, electric, or fluidic fields must be considered together with the reported numerical values.

To complement the cross-platform comparison in Table 1, Table 2 quantifies how representative optical, material, structural, and operating parameters modify actuator output. The reported data reveal bounded operating windows rather than unlimited gains. Increasing optical power generally accelerates heating and motion until insufficient relaxation, reduced deformation, overheating, or material damage becomes dominant. Photothermal-filler content is similarly non-monotonic, because stronger absorption can be offset by increased stiffness, aggregation, and reduced optical penetration. Geometry and transport length also regulate apparent kinetics: lattice architectures and oriented-chain actuation reduce the effective thermal or solvent-transport limitation, whereas bulk diffusion slows centimetre-scale bending. Wavelength-selective absorbers and pulsed irradiation provide additional control over spatial addressability and gait, but their benefits depend on wavelength matching, medium temperature, pulse duration, and recovery time. Performance should therefore be reported as a parameter-dependent operating envelope—including threshold, optimum, and failure regimes—rather than as a single maximum value.

## 8. Future Perspectives

Future research should move light-responsive hydrogel robots from demonstrations of isolated deformation toward reproducible systems capable of performing defined tasks. At the material level, wavelength-selective networks that combine photothermal absorbers, molecular photoswitches, and reversible chemical interactions could separate locomotion from shape programming, adhesion control, or cargo release while reducing unnecessary thermal exposure. Material selection should therefore consider optical efficiency together with reversibility, fatigue resistance, component stability, biocompatibility, and the light dose required to generate a specified mechanical output. At the structural level, validated multiphysics models integrating optical propagation, heat and solvent transport, network mechanics, interfacial contact, and fluid–structure interaction should be combined with three-dimensional printing and laser microfabrication to relate illumination patterns directly to deformation and trajectory. This approach could replace empirical shape selection with task-oriented structural design while improving manufacturing reproducibility. Further progress will also require intrinsic sensing and closed-loop control so that light position, intensity, and exposure time can be adjusted according to the robot’s actual deformation, temperature, and location. For hybrid systems, magnetic, electric, or acoustic fields may provide long-range navigation, whereas light controls local deformation, adhesion, gripping, or release; however, the contribution of each input must be quantified to permit meaningful comparison. Biomedical translation should follow a staged route from flow models and three-dimensional cell systems to ex vivo tissues and application-specific animal models, with particular attention to optical penetration, thermal dose, degradation, immune response, and retrieval of persistent components. Ultimately, progress should be judged by reliable task completion under defined optical, mechanical, and environmental conditions rather than by deformation amplitude or locomotion speed alone.

## 9. Conclusions

This review examined light-responsive hydrogel soft robots across actuation mechanisms, fabrication methods, structural architectures, locomotion modes, and functional applications. The principal conclusion is that optical responsiveness alone does not determine robotic performance. Useful motion results from a connected sequence in which photon absorption or molecular switching changes the thermal or chemical state of the network, induces solvent redistribution and mechanical strain, and is subsequently converted by structural asymmetry and environmental interaction into directional displacement. The performance of a hydrogel robot must therefore be evaluated at the system level rather than solely through temperature change, swelling ratio, or bending amplitude.

Photothermal actuation offers broad material compatibility and effective remote activation, but its rate and stability remain dependent on optical penetration, heat dissipation, and water transport. Photochemical systems provide finer spatial or wavelength-selective regulation, although their performance may be limited by incomplete reversibility, chromophore fatigue, and restricted light penetration. Bilayers and continuous gradients provide relatively direct routes for converting differential swelling into bending, whereas anisotropic networks, programmable monolayers, topological bodies, and lattice structures enable more complex deformation and multimodal operation. Regardless of architecture, net locomotion requires spatial or temporal asymmetry produced through friction, patterned illumination, structural polarity, topology, self-shadowing, or fluid interaction. Hybrid systems extend navigation and task capability, but the mechanical contribution of light must be distinguished from propulsion supplied by magnetic, electric, or fluidic effects.

The studies reviewed here demonstrate that light-responsive hydrogels can support crawling, rolling, jumping, swimming, reconfiguration, self-sensing, cargo manipulation, localized cellular stimulation, and in vitro drug screening. Nevertheless, most systems remain laboratory prototypes, and the representative biomedical platforms have mainly been evaluated in artificial channels, cultured cells, and three-dimensional spheroids. Further development depends on reproducible fabrication, cyclic durability, standardized performance reporting, controlled optical and thermal exposure, reliable feedback, and appropriate evaluation of degradation or retrieval. Light-responsive hydrogels are therefore most effective as robotic materials when molecular response, structural mechanics, environmental coupling, and control strategy are designed together. The maturity of the field should ultimately be assessed by repeatable completion of defined tasks under relevant operating conditions rather than by isolated demonstrations of large deformation or high instantaneous speed.

## Figures and Tables

**Table 1:** Quantitative comparison of representative light-responsive hydrogel actuators and robotic systems.

Representative System and Primary Mechanism	Operating Conditions: Scale, Medium and Optical Input	Response and Quantitative Performance	Comparative Advantage and Principal Limitation	Ref.
Ag-NP/PNIPAM multi-joint microactuator; local photothermal deswelling	Microscale; water/substrate; 800 nm structured light, <10 mW	30 ms response; >10 modes; independent/linked joints; microparticle collection	Strength: rapid, low-power and addressable. Limit: TPP/photoreduction, fixed substrate and clear optical path required.	[69]
GO-infiltrated PNIPAm-4HBA crawler; asymmetric bend/recovery	23 × 2 × 2 mm; air/glass; 808 nm, 7.1 W	20 mm in 10 s; ≈2.0 mm s^−1^; v* ≈ 0.087 BL s^−1^; pushes 130 mg by 8 mm in 39 s	Strength: large displacement with a simple body. Limit: high power and contact dependence; standardized force/cycle data NR.	[82]
Fe_3_O_4_-PNIPAm bilayer crawler; ratchet-assisted photothermal bending	54 mm; air/ratchet; broadband, 5000 W m^−2^ in the response test	30° in 300 s; recovery ≈ 360 s; 54.5 mm h^−1^ (v* ≈ 2.8 × 10^−4^ BL s^−1^); ≈10 mm cycle^−1^	Strength: simple, robust bilayer. Limit: slow hydration cycle; added water and ratchet required; illumination reports differ.	[83]
PNIPAM-SWNT lattice microrobot; scanned photothermal peristalsis	≈100 × 50 × 20 μm; aqueous substrate; scanning laser, 50–60 mW, 10–14 Hz	30.58% shrinkage in 1.46 s; 15.15 μm s^−1^ (0.14 BL s^−1^); rotation 29.38° s^−1^; one-sixth energy vs. solid control	Strength: efficient multimodal motion and closed-loop navigation. Limit: focused scanning/vision required; overheating disrupts operation.	[80]
Fe_3_O_4_/poly(sodium acrylate) jumper/roller; vapour-bubble impulse	≈2 mm; air; 808 nm, 2.34 W (jump) or 0.67 W (roll)	0.8 s jump; 1.3 s roll; 1.6 m s^−1^ take-off; ≈15 cm height; ≈0.10 m s^−1^ roll; ≈4.05 mN	Strength: highest absolute speed; operates in air. Limit: high temperature, water loss and narrow power window; burning above 2.34 W.	[18]
Anisotropic cylindrical hydrogel; constant-light strain-gradient rolling	Millimetre scale; submerged surface; constant light, wavelength/power NR	1.27 mm s^−1^ horizontally; 0.57 mm s^−1^ on an 18° incline	Strength: continuous motion without light modulation. Limit: contact, illumination angle and underwater boundaries govern performance.	[88]
Au-coated PNIPAM tapered ribbon; coil–uncoil microswimming	80/120 μm; water, 30 μm channel; pulsed IR, power NR	Millisecond-pulse operation; ≈500 μm s^−1^; 6 BL s^−1^	Strength: highest body-length-normalized speed. Limit: tuned pulses and confinement required; boundary effects control direction.	[91]
Reprogrammable disulfide-PNIPAM robot; oriented-chain actuation	Millimetre–centimetre scale; water; UV programming plus periodic photothermal light	0.3–1.7 Hz oscillation; one body reprogrammed for swimming, crawling or rotation	Strength: rewritable anisotropy and multiple gaits. Limit: pre-deformation, UV programming and periodic illumination required.	[16]
MoO_2_/PNIPAM-alginate gradient actuator; actuation plus sensing	1 mm strip; water; 808 nm, 2 W cm^−2^	Heating 3.7 °C s^−1^; sensing 140 ms; GF 3.94 to 600% strain; 336° at 21° s^−1^ in 50 °C water; grasp 15–20 s	Strength: integrated actuation and sensing. Limit: peak bending rate was not measured under NIR; resistance also depends on temperature/hydration.	[58]
Fe_3_O_4_-protein hydrogel robot; light-controlled adhesion with magnetic navigation	Centimetre scale; water/artificial vessel; IR plus magnetic field	Soft–stiff switching in 9 s per 10 °C; adhesion 30–40 kPa; cargo transport and artificial-vessel sealing	Strength: combines adhesion, morphing and manipulation. Limit: magnetic propulsion; vessel repair demonstrated only in an artificial model.	[101]
TACSI microrobot; photothermofluidic motion and thermometry	Microscale; culture medium; 785 nm, 2–6 μW μm^−2^	37–57 °C in ≈100 ms; ≈2 °C resolution; 5–65 μm s^−1^; 72 h in-vitro test	Strength: mobility, local stimulation and temperature feedback. Limit: convection-driven rather than deformation-driven; in vitro only.	[104]

**Table 2:** Quantitative influence of representative optical, material, structural, and operating parameters on light-responsive hydrogel robot performance.

Key Parameter	Representative Range or Contrast	Quantified Response	Observed Trend and Design Implication	Ref.
MXene loading	0.3, 0.7 and 1.0% MXene in PNIPAM; white light at 25–70 mW cm^−2^	The 0.3% formulation gave the shortest time constant (74 s) and a 200% bending-angle change after 100 s at 70 mW cm^−2^; reversible for 10 cycles.	An intermediate loading outperformed 0.7 and 1.0%. Absorption, network stiffness and shrinkage must be optimized together; more absorber is not necessarily better.	[41]
Wavelength–absorber matching	Nd_2_O_3_/808 nm and Yb_2_O_3_/980 nm; 3 W cm^−2^; water at 28 °C	Matched pairs produced local transitions; mismatched pairs showed no response. Nd_2_O_3_ saturated within seconds, whereas Yb_2_O_3_ required ≈100 s.	Spectral matching enables independent addressing. The 980 nm response is slower because water absorbs more strongly, increasing propagation and thermal-loss constraints.	[42]
Laser power and motion threshold	808 nm: 0.04–0.39 W rolling only; 0.39–0.67 W mixed mode; 0.67–2.34 W jumping only	Jump speed rose from ≈0.02 to 1.6 m s^−1^ as power increased from 0.39 to 2.34 W; rolling rose from 0.44 to 10 cm s^−1^ over 0.04–0.67 W. Burning occurred above 2.34 W.	Speed increased nearly linearly within each regime, but power also selected the gait and defined a sharp damage limit.	[18]
Scanning power and frequency	30 mW versus 60 mW; scanning frequency from 4.65 to 14 Hz	At 30 mW, speed peaked at 2.22 μm s^−1^ at 4.65 Hz and was nearly zero at 10 Hz. At 60 mW, the optimum shifted to 10–14 Hz and speed reached 15.15 μm s^−1^.	Optical power determines the usable frequency window. Excessive scan frequency leaves insufficient time for heat accumulation and reduces displacement per cycle.	[80]
Lattice versus solid architecture	Lattice robot at 50 mW versus solid robot at 300 mW	Lattice shrinkage reached 30.58% in 1.46 s versus 10.55% in 2.50 s for the solid control; one-sixth of the laser energy produced approximately threefold the locomotion speed.	Reducing thermal mass and transport distance improves deformation rate and energy use, although porous structures may reduce load-bearing capacity.	[80]
Oscillation frequency	Periodic photothermal operation at 0.3–1.7 Hz	Programmed hydrogels produced swimming, stepwise crawling and rotation; increasing frequency shortened each cycle but reduced actuation amplitude because full transformation was not completed.	Bypassing bulk water diffusion enables high frequency, but speed and deformation amplitude remain coupled and cannot be maximized simultaneously.	[16]
Pulse and recovery timing	Pulsed IR actuation of 80 or 120 μm ribbons in a 30 μm-high channel	An optimized pulse sequence produced ≈500 μm s^−1^ (6 BL s^−1^); changing pulse and recovery intervals shifted the behaviour between forward swimming and local spinning.	Net propulsion requires a non-time-reversible heat–relaxation cycle. Both insufficient recovery and excessive delay reduce useful translation.	[91]
Ionic conditioning and thermal state	CaCl_2_-treated versus untreated gradient hydrogel; 50 °C bath; 808 nm irradiation	CaCl_2_ treatment increased actuation speed 3.4-fold; the treated gel bent 336° at 21° s^−1^ in 50 °C water. NIR heating reached 3.7 °C s^−1^; sensing response was 140 ms.	Ionic conditioning lowers the transition temperature and accelerates deswelling. Thermal-bath bending and NIR heating metrics must be reported separately.	[58]
Crosslinking degree and temperature	0, 5, 15 and 30% di-tyrosine pre-crosslinking; temperature-controlled adhesion	The 15 and 30% gels showed transition temperatures of 43.5 and 52.7 °C. A 10 °C change switched the selected gel within 9 s; underwater adhesion reached 30–40 kPa.	Higher pre-crosslinking improves room-temperature cohesion/adhesion but raises the temperature required for activation, creating a strength–accessibility trade-off.	[101]

## Data Availability

No new data were created or analyzed in this study. Data sharing is not applicable to this article.
